# Inhibition of ALOX12–12-HETE Alleviates Lung Ischemia–Reperfusion Injury by Reducing Endothelial Ferroptosis-Mediated Neutrophil Extracellular Trap Formation

**DOI:** 10.34133/research.0473

**Published:** 2024-09-12

**Authors:** Chongwu Li, Peigen Gao, Fenghui Zhuang, Tao Wang, Zeyu Wang, Guodong Wu, Ziheng Zhou, Huikang Xie, Dong Xie, Deping Zhao, Junqi Wu, Chang Chen

**Affiliations:** ^1^ Department of Thoracic Surgery, Shanghai Pulmonary Hospital, School of Medicine, Tongji University, Shanghai, China.; ^2^ Shanghai Engineering Research Center of Lung Transplantation, Shanghai, China.; ^3^ Department of Thoracic and Cardiovascular Surgery, The First Affiliated Hospital of Chongqing Medical University, Chongqing, China.; ^4^ Department of Pathology, Shanghai Pulmonary Hospital, School of Medicine, Tongji University, Shanghai, China.

## Abstract

Lung ischemia–reperfusion injury (IRI) stands as the primary culprit behind primary graft dysfunction (PGD) after lung transplantation, yet viable therapeutic options are lacking. In the present study, we used a murine hilar clamp (1 h) and reperfusion (3 h) model to study IRI. The left lung tissues were harvested for metabolomics, transcriptomics, and single-cell RNA sequencing. Metabolomics of plasma from human lung transplantation recipients was also performed. Lung histology, pulmonary function, pulmonary edema, and survival analysis were measured in mice. Integrative analysis of metabolomics and transcriptomics revealed a marked up-regulation of arachidonate 12-lipoxygenase (ALOX12) and its metabolite 12-hydroxyeicosatetraenoic acid (12-HETE), which played a pivotal role in promoting ferroptosis and neutrophil extracellular trap (NET) formation during lung IRI. Additionally, single-cell RNA sequencing revealed that ferroptosis predominantly occurred in pulmonary endothelial cells. Importantly, *Alox12*-knockout (KO) mice exhibited a notable decrease in ferroptosis, NET formation, and tissue injury. To investigate the interplay between endothelial ferroptosis and NET formation, a hypoxia/reoxygenation (HR) cell model using 2 human endothelial cell lines was established. By incubating conditioned medium from HR cell model with neutrophils, we found that the liberation of high mobility group box 1 (HMGB1) from endothelial cells undergoing ferroptosis facilitated the formation of NETs by activating the TLR4/MYD88 pathway. Last, the administration of ML355, a targeted inhibitor of Alox12, mitigated lung IRI in both murine hilar clamp/reperfusion and rat left lung transplant models. Collectively, our study indicates ALOX12 as a promising therapeutic strategy for lung IRI.

## Introduction

Lung transplantation is the most effective treatment for patients with end-stage lung diseases. However, primary graft dysfunction (PGD), which is caused mainly by ischemia–reperfusion injury (IRI), persists as a pivotal and prevalent posttransplant complication, and is closely linked to early and late mortality [1,2]. Unfortunately, no treatment has been approved for IRI after lung transplantation. Recent research has indicated that cell death and inflammation are the most important signals during IRI [3]; thus, it is essential to focus on addressing cell death and inflammation for preventing and treating lung IRI.

Ferroptosis is a recently recognized form of cell death, and its main characteristic is iron-dependent lipid peroxidation [4]. Despite its known significance in multiple disorders like hepatic and myocardial IRI [5–7], its implication in lung IRI remains inadequately explored. The foundation of ferroptosis is lipid metabolism, in which polyunsaturated fatty acids like arachidonic acid (AA) undergo oxidation to drive the cell death process [8]. The arachidonate lipoxygenase (ALOX) family, which are key enzymes involved in AA metabolism, participate in generating lipid hydroperoxides. These hydroperoxides serve as crucial substrates in the Fenton reaction during ferroptosis [9]. Previous reports have demonstrated that ALOX12 is essential for the p53-dependent induction of ferroptosis in tumor suppression and promotes mitochondrial lipid peroxidation in liver IRI [10,11] and that ALOX15 can promote cardiomyocyte ferroptosis during myocardial IRI [6]. However, whether the ALOX family participates in the modulation of ferroptosis in lung IRI remains unclear.

Sterile inflammation triggered by programmed cell death is another feature of lung IRI, which is regulated mainly by neutrophils [12,13]. One highly effective role of neutrophils is their ability to generate neutrophil extracellular traps (NETs). NETs can ensnare and eliminate bacteria, fungi, parasites, and viruses, but if dysregulated, they can also cause tissue injury [14]. It has been shown that NETs contribute to lung injury, cause PGD, and prevent allograft tolerance after lung transplantation in animal models [15,16]. Moreover, NETs detected in ex vivo lung perfusion (EVLP) perfusate correlated with early posttransplant outcome clinically [17]. However, the interplay between programmed cell death and NETs in lung IRI remains elusive.

In the present study, we observed an up-regulation of ALOX12 and its derivative, 12-hydroxyeicosatetraenoic acid (12-HETE), following lung IRI, which facilitates ferroptosis in lung endothelial cells. Moreover, the subsequent release of high mobility group box 1 (HMGB1) during ferroptotic cell death caused neutrophil infiltration and NET formation by activating the TLR4/MYD88 pathway. Notably, genetic or pharmacological inhibition of ALOX-12-HETE signaling alleviated lung IRI in both a murine model with hilar occlusion and a rat orthotopic lung transplant model.

## Results

### ALOX12 and 12-HETE were up-regulated after lung IRI

**Fig. 1.**
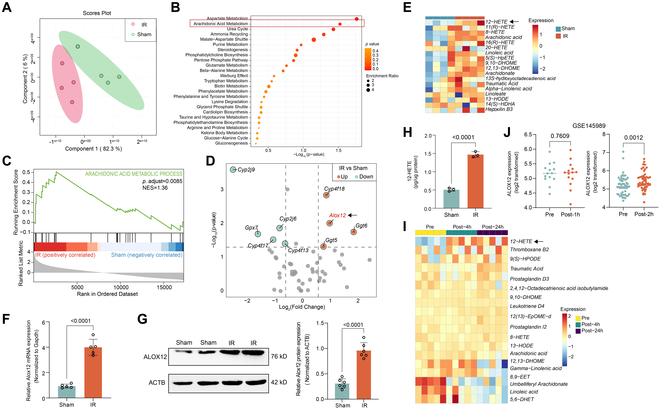
ALOX12 and12-HETE were up-regulated after lung ischemia–reperfusion injury (IRI). (A) Partial least squares discriminant analysis (PLS-DA) of the metabolomics data demonstrated distinct metabolic profiles between the sham and IRI groups; *n* = 4 in each group. (B) KEGG pathway enrichment analysis identified AA metabolism pathway as significantly enriched in IRI compared to sham. The top 25 enriched pathways are shown. (C) GSEA of the RNA-Seq data revealed that AA metabolism was enriched in the IRI group; *n* = 3 in each group. (D) Volcano plot showing differential gene expression in the AA metabolism pathway between the sham and IRI groups, with *Alox12* (arrow) among the most up-regulated genes; *n* = 3 in each group. (E) Heatmap of metabolites in the AA metabolism pathway showing that 12-HETE (arrow) was the metabolite with the greatest increase after lung IRI; *n* = 4 in each group. (F to H) Increased ALOX12 mRNA (F; *n* = 5 per group), protein (G; *n* = 6 per group), and 12-HETE (H; *n* = 3 per group) levels confirmed in lung tissue by qPCR, Western blot, and ELISA. (I) Metabolomics analysis of plasma from lung transplant patients corroborates the mouse model findings, with a marked increase in 12-HETE (arrow) levels after transplantation; *n* = 5 in each group. (J) Analysis of a public dataset (GSE145989) showing unchanged *ALOX12* expression in human lung tissues 1 h after lung transplantation (left panel, *n* = 14 per group), with a significant increase observed 2 h after transplantation (right panel, *n* = 53 per group). The data are presented as means ± SDs. Significance was examined by unpaired 2-sided *t* test in (F) to (H) and matched-pairs Mann–Whitney test in (J).

To examine the metabolic profiles after lung IRI, we first performed nontargeted metabolomics analyses of left lung tissue samples obtained from mice that underwent sham surgery or left lung IRI (1 h of ischemia followed by 3 h of reperfusion) in a hilar clamp mouse model. The results showed that the sham and IRI groups presented distinct metabolic profiles (Fig. 1A). Kyoto Encyclopedia of Genes and Genomes (KEGG) pathway analysis of the differentially abundant metabolites between the 2 groups identified AA metabolism as one of the most enriched pathways (Fig. 1B). Moreover, we performed RNA sequencing (RNA-Seq) of left lung tissue samples from the sham and IRI groups. Gene set enrichment analysis (GSEA) also revealed that AA metabolism was enriched in the IRI group (Fig. 1C). Notably, among the genes involved in AA metabolism, *Alox12*, which mainly metabolizes AA to 12-HETE, was one of the most highly up-regulated genes (Fig. 1D). Additionally, 12-HETE was the metabolite with the greatest increase in abundance among the detected AA metabolites according to metabolomics analysis (Fig. 1E). Furthermore, through quantitative polymerase chain reaction (qPCR) and immunoblotting analyses, we verified notable increases in both the mRNA and protein expression of Alox12 in lung tissue following IRI (Fig. 1F and G). Additionally, the increase in 12-HETE in the left lung tissue after lung IRI was confirmed by enzyme-linked immunosorbent assay (ELISA) (Fig. 1H). To explore the human relevance of our findings, we also performed nontargeted metabolomics analysis of 5 pairs of plasma samples from patients before and after lung transplantation. Consistent with the results obtained from the mouse model, 12-HETE was the metabolite with the greatest increase in abundance in the AA pathway after 4 and 24 h (Fig. 1I). Moreover, a publicly available dataset (GSE145989) [18] containing gene expression data from 67 pre/posttransplant human lung tissue pairs demonstrated that *ALOX12* expression did not significantly change after 1 h of reperfusion (Fig. 1J, left panel) but was up-regulated after 2 h of reperfusion (Fig. 1J, right panel). Collectively, these results demonstrated that the ALOX12–12-HETE pathway, which is one of the ALOX family members involved in AA metabolism, was markedly up-regulated after lung IRI in both a mouse model and human lung transplant recipients.

To investigate whether AA metabolism and *ALOX12* up-regulation are also involved in other lung diseases, we utilized publicly available datasets containing transcriptomic data of patients with COVID-19 (GSE151764, GSE155241, and GSE182917), lung fibrosis (GSE53845 and the dataset from Reyfman et al. [19]), and sepsis-induced acute lung injury (GSE10474 and GSE66890). Although the AA metabolism pathway was enriched in these diseases, the up-regulation of *ALOX12* was not observed (Fig. S1A to N). Therefore, the up-regulation of *ALOX12* may be specific to lung IRI.

### Lung IRI and NET formation were attenuated in *Alox12*-KO mice

**Fig. 2.**
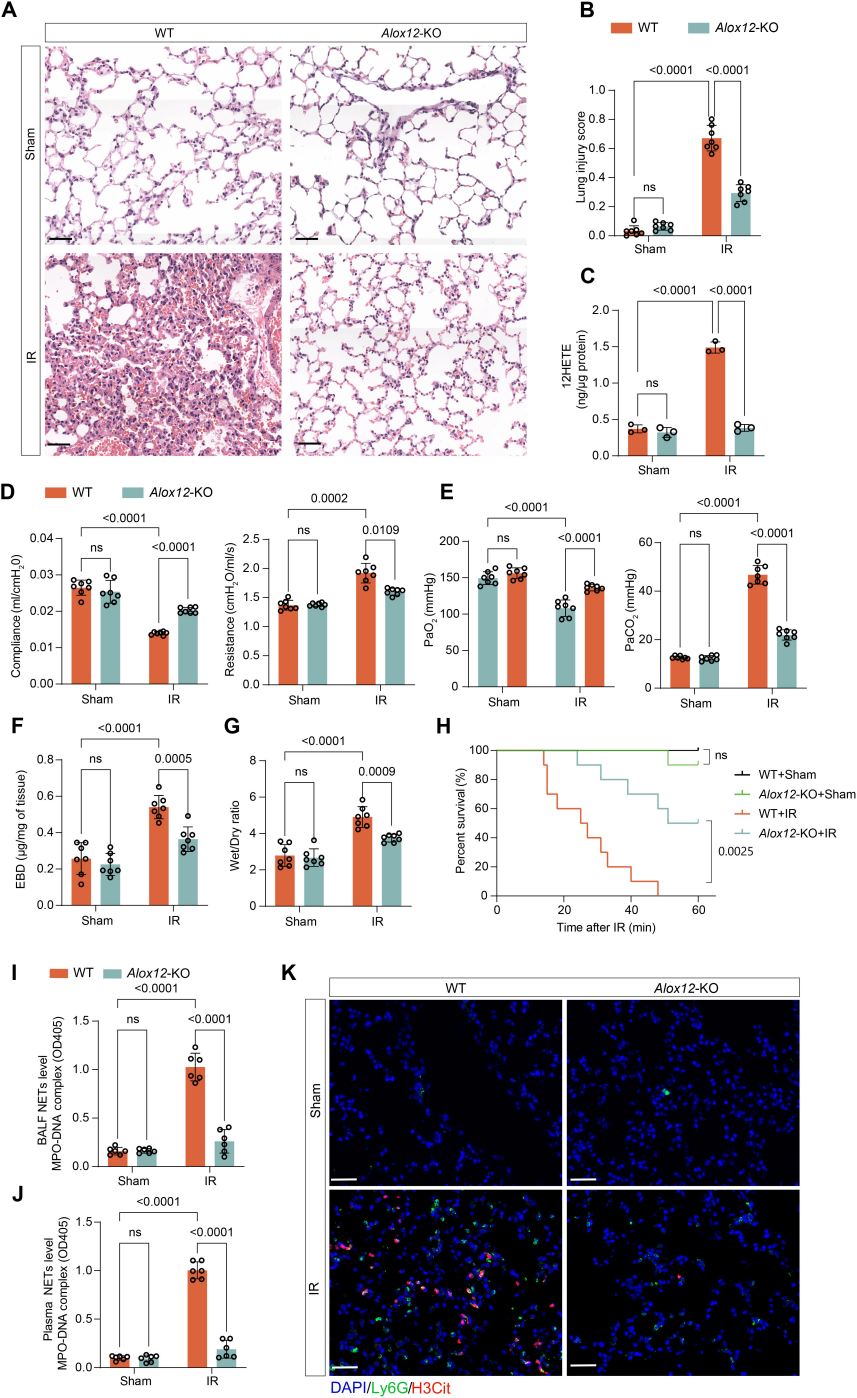
*Alox12*-KO alleviated lung injury, improved pulmonary function, prolonged survival, and reduced NET formation following lung IR. (A and B) H&E staining (A) and lung injury scores (B) showed significantly less lung damage in *Alox12*-KO mice compared to WT controls after IR. Scale bar, 50 μm; *n* = 7 in each group. (C) ELISA confirms a significant reduction in 12-HETE levels in the absence of *Alox12* after reperfusion; *n* = 3 in each group. (D and E) *Alox12*-KO mice exhibited better airway compliance, reduced airway resistance (D), higher PaO_2_, and lower PaCO_2_ (E) after IR, indicating preserved lung function; *n* = 7 in each group. (F and G) Evans blue dye extravasation (F) and wet/dry lung weight ratios (G) demonstrated reduced edema in *Alox12*-KO mice after reperfusion; *n* = 7 in each group. (H) Survival of mice depended solely upon the left lung (with right hilum ligated), showing that *Alox12*-KO mice had better survival rates compared with WT mice after lung IR; *n* = 10 in each group. (I to K) The level of NETs after reperfusion, measured by MPO-DNA complex in the BALF (I) and plasma (J), and immunofluorescence staining for H3Cit and Ly6G in lung tissues (K), was lower in *Alox12-*KO mice than in WT mice; *n* = 7 in each group. Scale bar, 50 μm. The data are presented as means ± SDs. Significance was examined with one-way ANOVA in (B) to (G), (I), and (J) and log-rank (Mantel–Cox) test in (H).

To further determine the function of *Alox12* in lung IRI, we established *Alox12*-knockout (KO) mice and subjected them to left lung ischemia and reperfusion (IR) in parallel with wild-type (WT) controls. Hematoxylin-eosin (H&E) staining of the lungs and the lung injury scores indicated a substantial decrease in lung damage in the *Alox12*-KO mice following IR (Fig. 2A and B). Additionally, there was a substantial reduction in the production of 12-HETE in *Alox12*-KO mice after reperfusion (Fig. 2C). Moreover, compared with WT mice, *Alox12*-KO mice exhibited a superior preservation of pulmonary function after lung IR, as evidenced by improved airway compliance and decreased airway resistance (Fig. 2D) and higher PaO_2_ and lower PaCO_2_ (Fig. 2E). Lung edema is another hallmark of lung IRI, and Evans blue dye analysis and wet/dry ratio measurements of the left lung tissues demonstrated that *Alox12*-KO mice had less microvascular permeability and pulmonary edema than WT mice after lung IR (Fig. 2F and G). Additionally, to better assess injured lung function, we ligated the right hilum after left lung reperfusion so that the mice depended solely upon the injured lung and determined the survival rate within 1 h, as previously reported [20]. We found that the survival rate of *Alox12*-KO mice is higher than that of WT mice after experiencing lung IR (Fig. 2H).

The acute inflammatory response is one of the most profound signals in lung IRI. Neutrophils, which are the driving force of the inflammatory process, have long been considered histological hallmarks and major causes of lung IRI [21]. NETs have been reported to be pathogenic in PGD after lung transplantation in both murine models and human lung transplant recipients [15]. Therefore, to assess local and systemic inflammation, we detected the levels of proinflammatory cytokines and the myeloperoxidase (MPO)-DNA complex (a marker of NETs) in bronchoalveolar lavage fluid (BALF) and plasma. *Alox12* deficiency reduced the release of interleukin-6 (IL-6), C-X-C motif chemokine ligand 1 (CXCL1), tumor necrosis factor-α (TNF-α), and granulocyte colony-stimulating factor (G-CSF) in BALF and plasma (Fig. S2A to H) after lung IR. Additionally, the increase in neutrophil infiltration into the lungs after reperfusion was prevented in *Alox12*-KO mice (Fig. S3), suggesting that *Alox12* deficiency could reduce neutrophil recruitment. Moreover, the increase in NET formation in the BALF and plasma after lung IR was inhibited in *Alox12*-KO mice (Fig. 2I and J). As expected, the same result was observed by immunofluorescence staining of NETs in lung tissue, and *Alox12* deficiency reduced the increase in citrullinated histone H3 (H3Cit)-Ly6G coexpression after lung IR (Fig. 2K). Collectively, these results suggest that *Alox12* is an essential driver of NET formation and lung IRI. Genetic inhibition of *Alox12* can reduce neutrophil-driven inflammation and alleviate lung injury.

### *Alox12* deficiency in mice inhibited ferroptosis during lung IRI

**Fig. 3.**
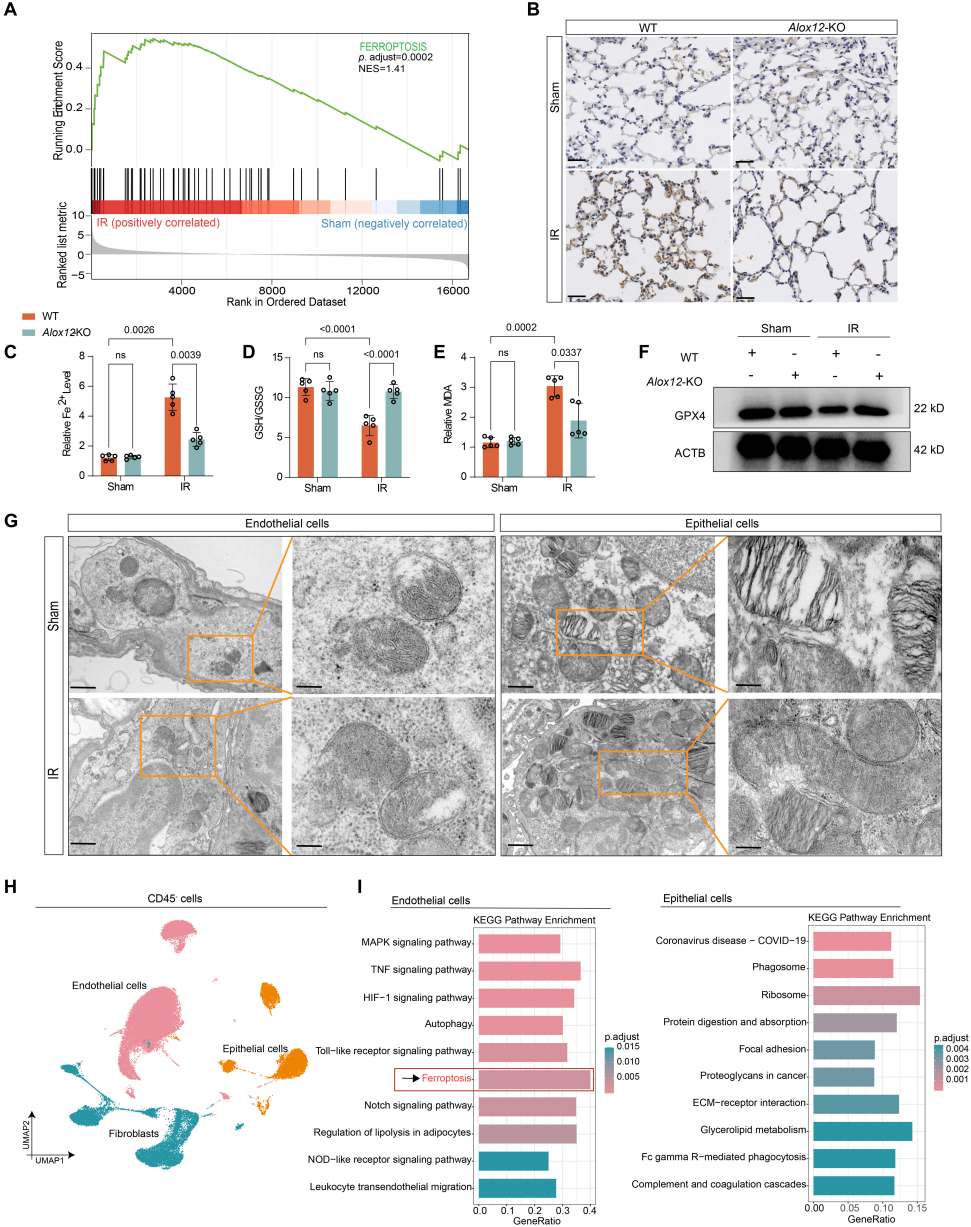
*Alox12*-KO inhibited IR-induced lung endothelial ferroptosis. (A) GSEA of RNA-Seq data showing the enrichment of ferroptosis signaling pathway in the IR group. (B) Representative images of Pear’s DAB staining revealed reduced ferric iron deposits in *Alox12*-KO mice after lung IR. Scale bar, 20 μm. (C to F) *Alox12*-KO mice presented reduced ferroptosis markers after lung IR compared to WT mice, including a lower Fe^2+^ level (C), an increased GSH/GSSG ratio (D), a reduced MDA level (E), and an elevated GPX4 protein level (F); *n* = 5 in each group. (G) Representative TEM images showing that the lung endothelial cells, but not the epithelial cells, displayed typical ferroptosis-associated mitochondrial morphologies after lung IRI. Scale bar, 1 μm in the left panel and 500 nm in the right panel. (H) Uniform manifold approximation and projection for dimension reduction (UMAP) plots displaying 50,599 CD45^−^ cells from left lung tissues separated into 3 main clusters; *n* = 3 in each group. (I) KEGG pathway enrichment analysis of differentially expressed genes between sham and IRI groups showed that the ferroptosis pathway was enriched in lung endothelial cells but not in epithelial cells. A Benjamini–Hochberg-corrected *P* value of ≤0.01 was considered significant. The data are presented as means ± SDs. Significance was examined by one-way ANOVA in (C) to (E).

Next, we examined the underlying mechanism by which *Alox12* deficiency reduces NET formation and lung IRI. Given that *Alox12* is a key regulator of ferroptosis [4], we hypothesized that inhibiting *Alox12* can alleviate ferroptosis in lung IRI. Consistent with previous reports [22], GSEA showed that the ferroptosis pathway was enriched after lung IR (Fig. 3A). Additionally, Perls’ 3,3′-diaminobenzidine (DAB) staining of lung tissue demonstrated that ferric iron deposits were increased after lung IR compared with that after sham surgery, and that the relative level of Fe^2+^ was increased (Fig. 3B and C). Moreover, the ratio of reduced glutathione (GSH) to oxidized glutathione [glutathione disulfide (GSSG)], which is a marker of oxidative stress, was markedly decreased, and the level of malondialdehyde (MDA), which is a marker of lipid peroxidation, was markedly increased after lung IR compared with those in sham mice (Fig. 3D and E). In addition, the protein level of glutathione peroxidase 4 (GPX4), which protects cells from membrane lipid peroxidation, was down-regulated after lung IR (Fig. 3F). Notably, after genetically inhibiting *Alox12* in mice, ferroptosis was dramatically inhibited compared with that in WT mice subjected to lung IR, as evidenced by decreases in ferric iron deposits, the levels of Fe^2+^ and MDA, and the increases in the GSH/GSSG ratio and the protein level of GPX4 (Fig. 3B to F). Taken together, these results suggest the involvement of ferroptosis in the development of lung IRI and that inhibiting *Alox12* can reduce ferroptosis and therefore alleviate lung IRI.

While an earlier study have elucidated the pivotal involvement of ferroptosis in lung IRI [22], the authors did not examine which kind of cells in the lung parenchyma experienced ferroptotic cell death after lung IR. By using transmission electron microscopy (TEM), we found that the mitochondria of lung endothelial cells exhibited typical morphologies associated with ferroptosis after lung IRI, including a reduction in mitochondrial cristae, outer mitochondrial membrane rupture, and a decrease in condensed mitochondrial membrane density, which was not observed in lung epithelial cells (Fig. 3G). In addition, we further performed single-cell RNA-Seq (scRNA-Seq) of CD45^−^ cells isolated from the left lungs of the mice in the sham surgery and lung IRI groups (*n* = 3 in each group). Transcriptomic data were obtained for 50,599 cells. Lung endothelial cells, epithelial cells, and fibroblasts were identified (Fig. 3H). KEGG pathway enrichment analysis of the differentially expressed genes between the sham and IRI groups demonstrated that ferroptosis was significantly enriched in endothelial cells only but not in epithelial cells (Fig. 3I). Furthermore, we sorted lung endothelial cells (CD45^−^CD31^+^) by fluorescence-activated cell sorting (FACS) from WT or *Alox12*-KO mouse lungs after sham surgery or IRI (Fig. S4A). Compared with those from the sham group, endothelial cells sorted from WT mouse lung tissues subjected to IRI exhibited increased levels of lipid peroxidation products (Fig. S4B and C), cell injury, (Fig. S4D), and MDA (Fig. S4E) and a decreased level of the GSH/GSSG ratio (Fig. S4F). This finding is consistent with the scRNA-Seq results, which showed that lung endothelial cells undergo ferroptosis during IRI. Additionally, ferroptosis was significantly inhibited in endothelial cells sorted from *Alox12*-KO mice after reperfusion (Fig. S4B to F). Collectively, these results indicated that lung endothelial cells experienced ferroptotic cell death during lung IRI.

### Knockdown of *ALOX12* in endothelial cells inhibited HR-induced ferroptosis in vitro

**Fig. 4.**
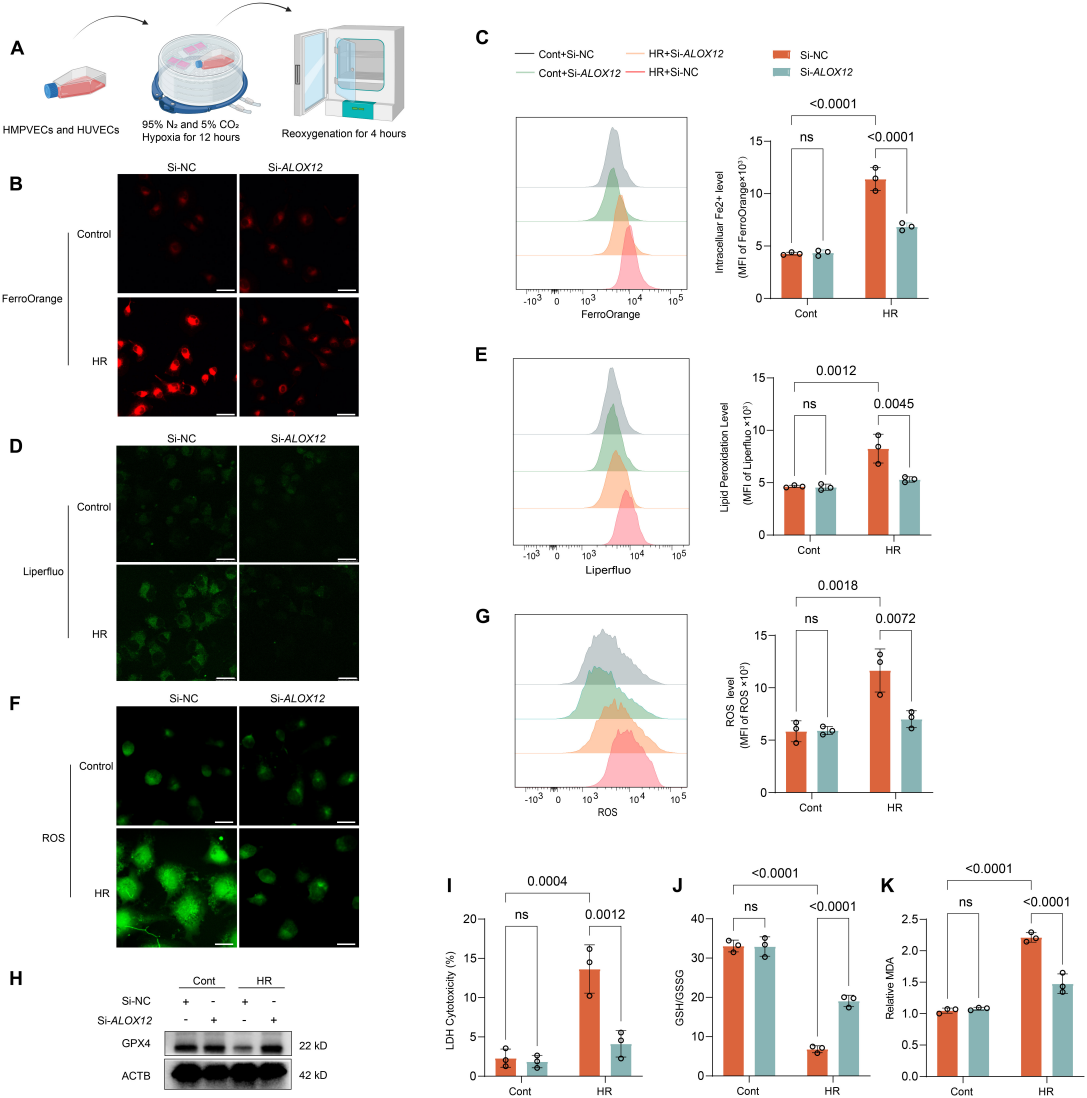
*ALOX12* knockdown in HPMVEC cells inhibited hypoxia/reoxygenation-induced ferroptosis in vitro. (A) Schematic diagram illustrating the hypoxia/reoxygenation model establishment. (B to G) *ALOX12* knockdown reduced HR-induced ferroptosis in HPMVEC cells, as evidenced by lower levels of iron, lipid ROS, and total ROS, measured via confocal microscopy (B, D, and F) and flow cytometry (C, E, and G) using FerroOrange, Liperfluo, and ROS Assay Kit staining, respectively [*n* = 3 per group; scale bars, 25 μm (B and D) and 20 μm (F)]. (H) Immunoblotting showed that HPMVEC cells treated with Si-*ALOX12* prevented the down-regulation of the GPX4 protein level induced by HR. (I) *ALOX12* knockdown protected against the HR-induced cell injury, as detected by LDH cytotoxicity assay; *n* = 3 in each group. (J and K) Changes in the markers of ferroptosis induced by HR, including the GSH/GSSG ratio (J) and the relative level of MDA (K), were prevented in HPMVEC cells with *ALOX12* knockdown; *n* = 3 in each group. The data are presented as means ± SDs. Significance was examined by one-way ANOVA in (C), (E), (G), and (I) to (K).

Since endothelial cells were the predominant ferroptotic cells during lung IRI in the mouse model, we further established a hypoxia/reoxygenation (HR) cell model (Fig. 4A) using human pulmonary microvascular endothelial cells (HPMVECs) and human umbilical vein endothelial cells (HUVECs). To examine the role of *ALOX12* in ferroptosis in vitro, *ALOX12* was knocked down in HPMVEC cells or HUVEC cells with small interfering RNA (siRNA; Fig. S5A). After 12 h of hypoxia followed by 4 h of reoxygenation, HPMVEC cells and HUVEC cells exhibited increases in iron levels (Fig. 4B and C and Fig. S5B and C), lipid peroxidation products (Fig. 4D and E and Fig. S5D and E), and reactive oxygen species (ROS) production (Fig. 4F and G and Fig. S5F and G), as detected by fluorescence microscopy and flow cytometry. Notably, these increases in ferroptosis induced by HR were prevented by *ALOX12* knockdown (Fig. 4B to G and Fig. S5B to G). Moreover, *ALOX12* knockdown protected against the HR-induced cell injury and prevented changes in the GSH/GSSG ratio and MDA levels in HPMVEC cells (Fig. 4I to K) and HUVEC cells (Fig. S5I to K). Moreover, the protein level of GPX4 was down-regulated after HR, but this effect was prevented by *ALOX12* knockdown in HPMVEC cells (Fig. 4H) and HUVEC cells (Fig. S5H). Overall, our results suggested that inhibiting *ALOX12* could reduce endothelial ferroptosis induced by HR in vitro.

### NET formation was dependent on ferroptosis during lung IRI

**Fig. 5.**
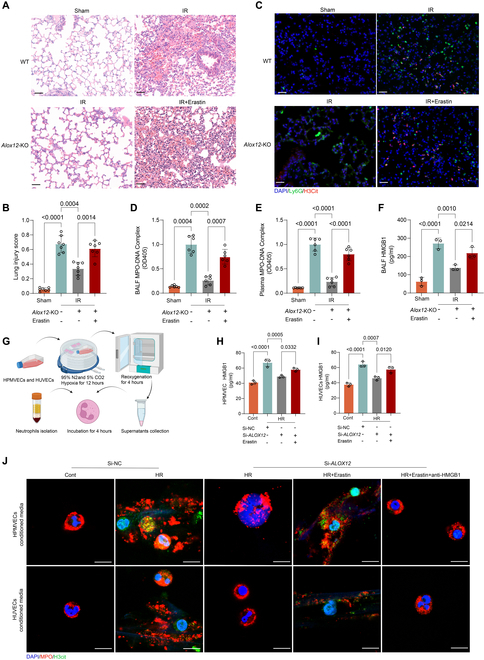
The HMGB1 released from ferroptotic cells contributed to NET formation in lung IRI. (A and B) H&E staining of left lung tissues (A) and lung injury scores (B) demonstrated that treatment with the ferroptosis inducer erastin reversed the protective effects seen in *Alox12*-KO mice after lung IR. Scale bar, 50 μm; *n* = 7 in each group. (C to E) Immunofluorescence staining for H3Cit and Ly6G in lung tissues (C), along with measurements of NET levels (MPO-DNA complex) in the BALF (D) and plasma (E), revealed that the reduction in NET formation in *Alox12*-KO mice was reversed by erastin treatment after reperfusion. Scale bar, 50 μm. *n* = 5 in each group. (F) The decrease in HMGB1 level detected in the BALF of *Alox12*-KO mice was reversed by erastin treatment after reperfusion; *n* = 5 in each group. (G) Schematic diagram of human circulating neutrophils incubated with supernatants of endothelial cells subjected to HR as conditioned medium. (H and I) The decrease in HMGB1 levels detected in the supernatants of HPMVEC cells (H) and HUVEC cells (I) with *ALOX12* deficiency after HR was reversed by erastin. *n* = 3 in each group. (J) Representative images of neutrophils stained with MPO and H3Cit after incubation with different conditioned medium showed that NET formation was dependent on endothelial ferroptosis and HMGB1 release in vitro. Scale bar, 10 μm. The data are presented as means ± SDs. Significance was examined by one-way ANOVA in (B) to (F), (H), and (I).

Next, we treated *Alox12*-KO mice subjected to lung IR with the ferroptosis inducer erastin and found that erastin treatment reversed the improvements in lung injury (Fig. 5A and B), pulmonary function (Fig. S6A and B), lung edema (Fig. S6C and D), and survival rate (Fig. S6E) in *Alox12*-KO mice after lung IR. In addition, the reduction in NET formation after lung IR by inhibiting ferroptosis via *Alox12* deficiency was reversed by erastin treatment, as evidenced by immunofluorescence staining of NETs in lung tissue (Fig. 5C) and MPO-DNA complex measurements in BALF (Fig. 5D) and plasma (Fig. 5E). Taken together, these data indicate that NET formation is dependent on ferroptosis during lung IRI.

### The HMGB1 released from ferroptotic endothelial cells contributed to NET formation

Next, we examined why inhibiting ferroptosis could reduce the formation of NETs in lung IRI. Previous reports have demonstrated that danger-associated molecular patterns (DAMPs), mainly HMGB1, are released from damaged cells to trigger sterile inflammation [23]. Moreover, HMGB1 can be released from ferroptotic cells and induce NET formation, and the level of HMGB1 is closely associated with PGD after lung transplantation [24–27]. Therefore, we hypothesized that endothelial cell ferroptosis contributed to NET formation via HMGB1 release during lung IRI. First, we detected the level of HMGB1 in the BALF of mice and found that HMGB1 was significantly increased after lung IRI but decreased by inhibition of *Alox12*, and erastin treatment reversed the changes in HMGB1 levels in *Alox12*-KO mice after lung IRI (Fig. 5F). Moreover, we collected the supernatants from HPMVEC cells and HUVEC cells subjected to HR, *ALOX12* knockdown, or erastin treatment, measured the level of HMGB1, and further incubated this conditioned medium with isolated circulating neutrophils (Fig. 5G). Consistent with the in vivo results, in the supernatants of HPMVEC cells and HUVEC cells, the level of HMGB1 was significantly increased after HR and was decreased by *ALOX12* knockdown, and this activity was abrogated by erastin treatment (Fig. 5H and I). After these supernatants were incubated with neutrophils, we found that, compared with that in the negative control, significantly greater NET formation was observed after stimulation with HR-conditioned medium from HPMVEC cells and HUVEC cells (Fig. 5J). More importantly, anti-HMGB1 reduced the NET formation induced by the conditioned medium from HPMVEC cells and HUVEC cells treated with erastin (Fig. 5J). Collectively, these results suggest that the HMGB1 released from ferroptotic endothelial cells contributes to NET formation during lung IRI.

### HMGB1 activated the TLR4-MYD88 pathway to mediate NET formation in lung IRI

**Fig. 6.**
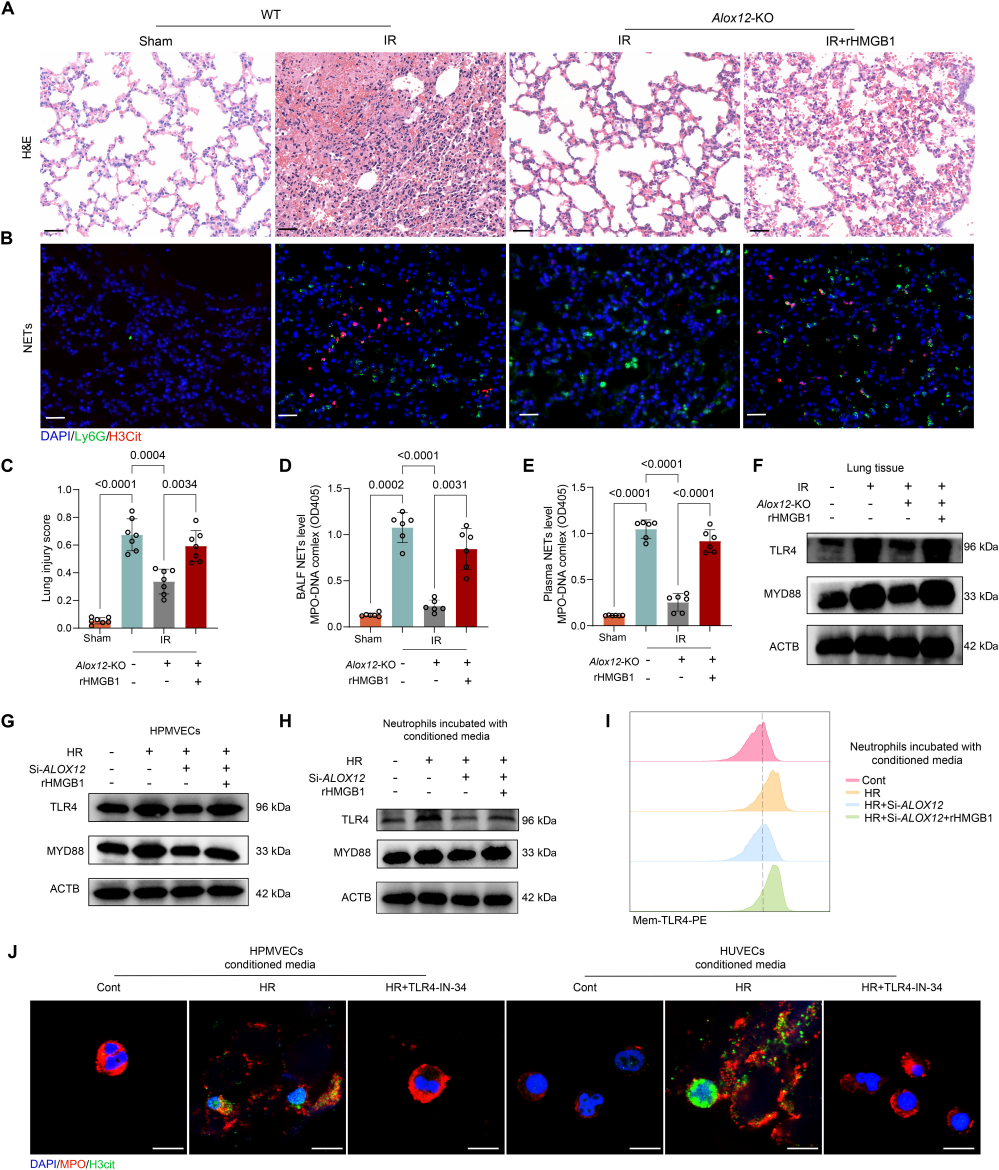
HMGB1 abrogated the protective effects of *Alox12* deficiency against IRI and activated the TLR4-MYD88 pathway to mediate NET formation. (A to E) rHMGB1 treatment reversed the inhibitory effects of *Alox12* deficiency on reperfusion-induced changes. H&E staining of left lung tissues (A) and lung injury scores (C) showed that the reduction in lung injury observed in *Alox12*-KO mice after reperfusion was reversed by the treatment with rHMGB1. Immunofluorescence staining for H3Cit and Ly6G in lung tissues (B), along with measurements of NET levels (MPO-DNA complex) in the BALF (D) and plasma (E), revealed that the reduction in NET formation in *Alox12*-KO mice after reperfusion was reversed by the treatment with rHMGB1. Scale bars, 50 μm (A) and (B); *n* = 7 in each group for (C) and *n* = 6 in each group for (D) and (E). (F and G) In both lung tissues after IRI (F), and HPMVEC cells subjected to HR (G), the protein levels of TLR4 and MYD88 increased following injury, but decreased with *Alox12* deficiency. This reduction was reversed by rHMGB1 treatment. (H and I) Immunoblotting and flow cytometry analyses revealed that both total protein levels of TLR4 and MYD88 (H), as well as TLR4 membrane levels (I) in neutrophils, increased after incubation with conditioned medium from HR-induced endothelial cells. These levels decreased with *ALOX12* knockdown but were restored by rHMGB1 treatment. (J) TLR4-IN-34, a TLR4 inhibitor, prevented NET formation in neutrophils exposed to conditioned medium from HR-treated endothelial cells. Scale bar, 10 μm. The data are presented as means ± SDs. Significance was examined by one-way ANOVA in (C) to (E).

To further confirm the critical roles of HMGB1 in lung IRI and NET formation, we treated *Alox12*-KO mice subjected to lung IRI with the recombinant HMGB1 (rHMGB1) protein. Notably, rHMGB1 treatment exacerbated lung injury in *Alox12*-KO mice after lung IR (Fig. 6A), as evidenced by increases in lung injury scores (Fig. 6C), airway resistance, PaCO_2_, microvascular permeability, and pulmonary edema and decreases in airway compliance, PaO_2_, and survival (Fig. S6A to F). Moreover, NET formation in lung tissue (Fig. 6B), plasma (Fig. 6D), and BALF (Fig. 6E) increased after rHMGB1 treatment.

Since the Toll-like receptor (TLR) signaling pathway is enriched in endothelial cells subjected to lung IRI and previous reports have shown that HMGB1 can activate TLR4 and its downstream MYD88 in endothelial cells and neutrophils to recruit and activate neutrophils, respectively [23,28,29], we hypothesized that the TLR4-MYD88 pathway was activated by HMGB1 to facilitate NET formation during lung IRI. First, we showed that the protein levels of TLR4 and MYD88 in lung tissue were markedly increased after lung IR (Fig. 6F). *Alox12* deficiency reduced the protein levels of TLR4 and MYD88, and this effect was reversed by rHMGB1 treatment (Fig. 6F). A similar outcome was observed in HPMVEC cells in vitro. rHMGB1 treatment also increased the protein levels of TLR4 and MYD88 in *ALOX12*-knockdown endothelial cells exposed to HR (Fig. 6G). Given that the effect of HMGB1 on the TLR4 pathway is not cell specific and is indispensable for neutrophil activation, we further measured the protein levels of TLR4 and MYD88 in neutrophils incubated with the conditioned medium of different endothelial cells. Notably, the total and membrane protein levels of TLR4 and MYD88 in neutrophils were significantly increased after incubation with HR-conditioned medium and were reduced after incubation with conditioned medium from *ALOX12*-knockdown endothelial cells exposed to HR; notably, these effects were reversed by rHMGB1 treatment (Fig. 6H and I). Additionally, NET formation induced by the conditioned medium from HPMVEC cells and HUVEC cells exposed to HR could be inhibited by TLR4-IN-34, a specific TLR4 inhibitor (Fig. 6J). Collectively, these findings indicate that the HMGB1 released from ferroptotic endothelial cells can activate the TLR4-MYD88 pathway to mediate NET formation.

### Pharmacological inhibition of ALOX12 reduced ferroptosis and NET formation and alleviated lung IRI

**Fig. 7.**
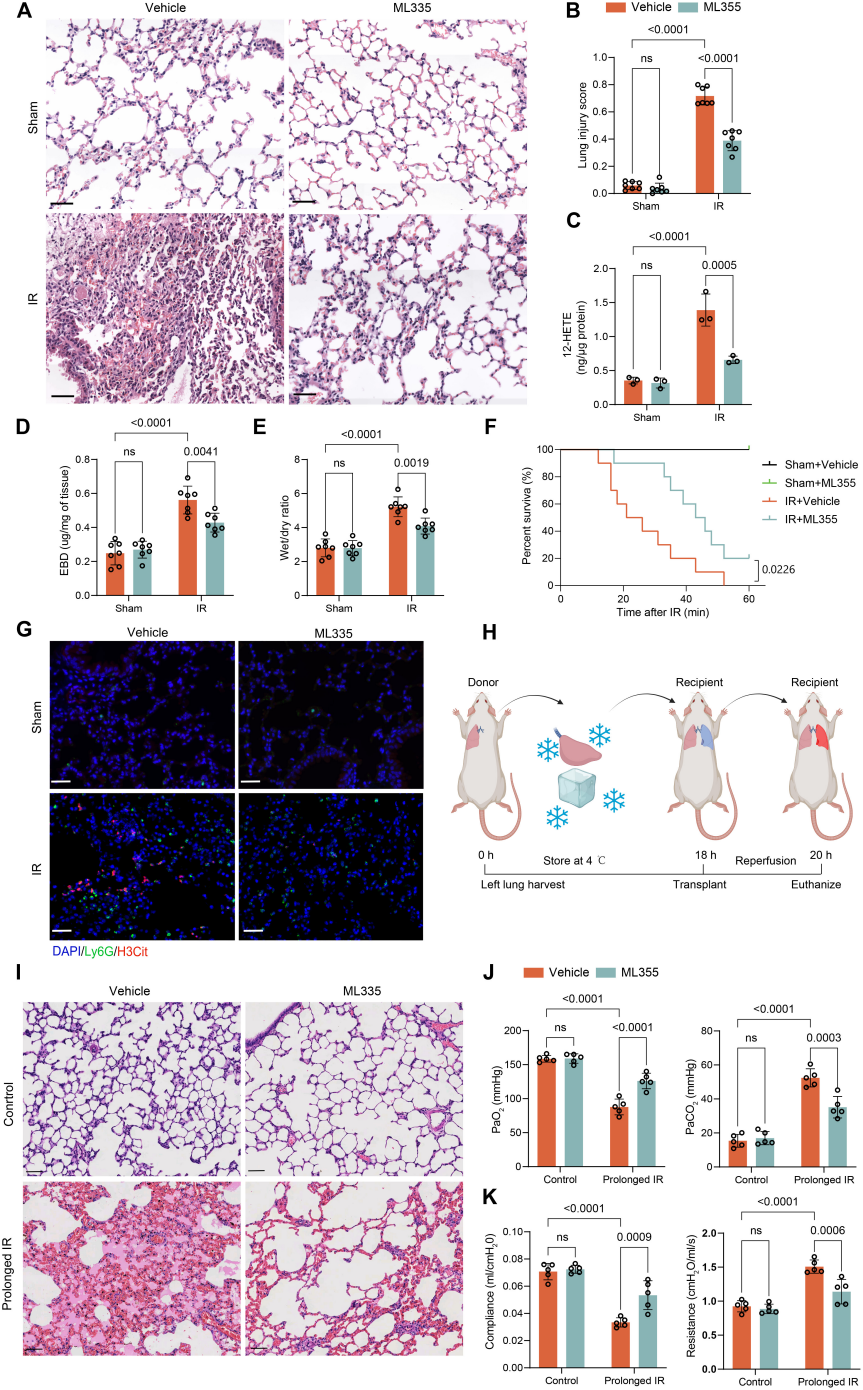
Pharmacological inhibition of ALOX12 by ML355 alleviated lung IRI in both the hilar clamp mouse model and the orthotopic left lung transplantation rat model. (A and B) H&E staining (A) and lung injury scores (B) showed that ML355 treatment reduced lung injury in the hilar clamp mouse model. Scale bar, 50 μm; *n* = 7 in each group. (C to E) ML355 treatment reduced 12-HETE production (C), pulmonary microvascular permeability (D), and pulmonary edema (E) after reperfusion in the hilar clamp mouse model; *n* = 3 in each group for (C) and *n* = 7 in each group for (D) and (E). (F) Survival analysis in mice depended solely upon the left lung with right hilum ligated, showing that ML355 improved survival after lung IR. *n* = 10 in each group. (G) Immunofluorescence staining of H3Cit and Ly6G revealed that ML355 reduced NET formation during lung IRI. Scale bar, 50 μm. (H) Schematic diagram of the orthotopic left lung transplantation rat model with prolonged cold ischemia time. (I to K) In the orthotopic left lung transplantation rat model with prolonged cold ischemia, ML355 alleviated lung injury, as shown by H&E staining of left lung tissues (I), and preserved pulmonary function, as measured by PaO_2_ and PaCO_2_ (J) and airway compliance and airway resistance (K). Scale bar, 50 μm; *n* = 5 in each group. The data are presented as means ± SDs. Significance was examined by one-way ANOVA in (B) to (E), (J), and (K) and log-rank (Mantel–Cox) test in (F).

Given the pivotal involvement of ALOX12 in lung IRI, pharmacological inhibition of ALOX12 emerges as a potential and promising treatment for posttransplantation IRI. Therefore, the ALOX12-specific inhibitor ML355, which was widely utilized in previous studies [30,31], was used to treat mice before the hilar clamp procedure. Notably, ML355 treatment alleviated histological lung injury (Fig. 7A and B), and the increase in 12-HETE levels induced by IR was also inhibited by ML355 (Fig. 7C). Additionally, ML355 treatment improved pulmonary function (Fig. S7A and B), reduced lung microvascular permeability (Fig. 7D) and pulmonary edema (Fig. 7E), and prolonged the survival of mice subjected to lung IR (Fig. 7F). In addition, ML355 inhibited ferroptosis induced by lung IR, as characterized by significant decreases in the levels of Fe^2+^ and MDA and increases in the GSH/GSSG ratio and GPX4 protein expression (Fig. S7C to F). Moreover, NET levels in lung tissue (Fig. 7G), plasma, and BALF (Fig. S7G) were reduced by ML355 treatment after reperfusion.

Although the hilar clamp mouse model has been widely used to study lung IRI, the orthotopic lung transplantation (OLT) model is more relevant to human PGD [32]. Thus, we established a left OLT rat model as previously reported [33]. Briefly, the left donor lung was harvested and preserved at 4 °C for 18 h before transplantation, followed by 2 h of warm reperfusion before the rats were euthanized (Fig. 7H). Consistent with a previous study [15], severe allograft injuries, including neutrophil infiltration, alveolar edema, intra-alveolar hemorrhage, and hyaline membrane development, were observed in this prolonged cold IR model (Fig. 7I). As observed in the mouse hilar clamp model, ML355 treatment significantly alleviated the lung injury (Fig. 7I and H) and pulmonary edema (Fig. S7I) induced by prolonged cold IR. Additionally, pulmonary function after lung IR was better preserved by ML355 treatment (Fig. 7J and K). Furthermore, NET formation after reperfusion was inhibited in the rats treated with ML355 (Fig. S7J).

## Discussion

In the present study, we used integrative metabolomics and transcriptomic analysis of human plasma and mouse lung tissue and showed that the ALOX12–12-HETE pathway, which is a key pathway in AA metabolism and an important regulator of ferroptosis, was up-regulated after lung IRI. Furthermore, scRNA-Seq and TEM revealed that endothelial cells underwent ferroptosis after lung IRI, which could be inhibited by *ALOX12* genetic deficiency. Subsequently, in the lung IRI mouse model and HR cell model, our findings demonstrated that the release of HMGB1 from endothelial cells undergoing ferroptosis facilitated NET formation through activation of the TLR4-MYD88 pathway. Finally, the administration of the ALOX12-specific inhibitor ML355 alleviated lung IRI by mitigating endothelial ferroptosis-induced NET formation. Importantly, in an OLT rat model with prolonged cold ischemia time that is more relevant to human PGD, ML355 ameliorated lung injury, alveolar edema, and NET formation. These results indicate that ALOX12 is a promising therapeutic target for lung IRI.

The ALOX family has recently been shown to be deeply involved in hepatic and myocardial IRI. Ma et al. [34] demonstrated that ALOX15 expression was dramatically up-regulated during ischemia-induced phospholipid peroxidation and increased susceptibility to ferroptosis during ischemia-induced myocardial damage. Cai et al. [6] showed that ALOX15 and 15-hydroperoxyeicosatetraenoic acid (15-HpETE) was significantly increased and promoted cardiomyocyte ferroptosis during the prolonged reperfusion phase. However, in our study, we did not observe the up-regulation of ALOX15 or its metabolites. Although Zhang et al. [35,36] reported that the ALOX12–12-HETE pathway was a key mediator of hepatic and myocardial IRI, the underlying mechanisms were different, and ferroptosis was not investigated. In hepatic IRI, the binding of 12-HETE to GPR31 triggers an inflammatory response, further intensifying liver damage [35], whereas in myocardial IRI, ALOX12 drives cardiomyocyte injury by suppressing adenosine monophosphate-activated protein kinas (AMPK) signaling [36]. Collectively, these studies reflect the organ specificity of IRI. In the present study, by using *Alox12-*KO mice, we revealed for the first time that ALOX12 was an important mediator of ferroptosis during lung IRI, reinforcing the critical roles of AA metabolism and the ALOX family in IRI.

Previously, only a few reports have shown that ferroptosis plays a significant role in lung IRI [22,37]. However, these reports did not investigate which cell type underwent ferroptosis during this procedure but used a human bronchial epithelial cell line (BEAS-2B) as an in vitro model. Using scRNA-Seq, we demonstrated for the first time that ferroptosis occurred mainly in lung endothelial cells in this model; thus, it is inappropriate to use lung epithelial cells to establish an HR model in vitro. Therefore, endothelial cells were used in the present study instead. Nevertheless, due to the relatively small number of genes enriched in the ferroptosis pathway, our analysis warrants further validation. It is still unclear why endothelial cells are more susceptible to ferroptosis in this lung IRI model. Notably, a recent study comparing the transcriptomics of BEAS-2B cells and HPMVEC cells after cold preservation and warm reperfusion in vitro showed that epithelial cells and endothelial cells have markedly distinct phenotypic transcriptomic signatures [38]. Therefore, it is reasonable that epithelial cells and endothelial cells undergo different types of cell death after lung IRI.

Neutrophils have long been considered the histological hallmark and major cause of lung IRI. Sayah and colleagues [15] reported that in the hilar clamp mouse model, OLT after prolonged cold ischemia mouse model, and in human lung transplant recipients with PGD, the level of NETs significantly increased. Intrabronchial administration of deoxyribonuclease-I (DNase-I) to disrupt NETs can reduce lung injury. However, Scozzi et al*.* [16] showed that the disruption of NETs by DNase-I could release NET fragments and induce innate immune responses that prevented lung transplant tolerance. These observations suggest NETs as a therapeutic target. In the present study, we demonstrated that a specific inhibitor of Alox12, ML355, effectively inhibited NET formation and lung IRI. ML355 has been proven safe in large animal models, including pigs and monkeys [36]. Recently, it has passed a phase 1a clinical trial for treating heparin-induced thrombocytopenia with good tolerability and no serious adverse events. It has also received a fast-track designation by the Food and Drug Administration [39], and a phase 2 clinical trial is ongoing (NCT05785819). Therefore, ML355 holds great promise for clinical use in the treatment of lung IRI.

The innate immune system can sense DAMPs released from damaged or dying cells to promote sterile inflammation [23]. Previous reports have demonstrated that HMGB1 can be released from injured hepatocytes to activate NET formation during liver IRI [29,40], which is similar to its effects on lung IRI observed in our study, although the specific type of cell death may be different. Recently, Li et al. [28] showed that ferroptotic cell death could initiate neutrophil recruitment through endothelial TLR4/Trif signaling to cause sterile inflammation in heart transplantation. However, whether neutrophil activation, especially NET formation, is present remains unexamined. In the present study, we further revealed that the neutrophil TLR4/MYD88 pathway was activated to promote NET formation during lung IRI. It is well established that HMGB1-TLR4 plays a critical role in NET formation [41], but how TLR4 expression is modulated remains unclear. Recently, Luo et al. [42] demonstrated that lipopolysaccharide (LPS) treatment increased *METTL3* expression in neutrophils, which initiates m6A modifications of *TLR4* mRNA to promote its protein expression and subsequently contribute to neutrophil activation. Further studies are needed to examine whether HMGB1 has a similar regulatory pattern.

The hilar clamp mouse model has been widely used to investigate the pathophysiological processes of lung IRI [32]. However, only warm ischemia occurs in this model. Therefore, we established an OLT rat model with prolonged cold ischemia that can simulate most aspects of lung transplantation [32].

In conclusion, we revealed that ALOX12-12-HETE was up-regulated and contributed to endothelial ferroptosis. Moreover, HMGB1 was released from endothelial cells undergoing ferroptosis to promote NET formation through activation of the TLR4/MYD88 pathway. Specifically targeting ALOX12 protected mice and rats from lung IRI. Taken together, our results show that targeting ALOX12 is a promising therapeutic strategy for lung IRI and PGD after lung transplantation.

## Materials and Methods

### Ethics statement

The Research Ethical Board of Shanghai Pulmonary Hospital (REB number: K22-223) approved the study protocol and the use of human samples, and informed consent for the collection of blood samples was obtained. Approval for all animal experiments was granted by the Animal Care and Use Committee of Shanghai Pulmonary Hospital (FKDS-22-1-067), adhering to the *Guide for the Care and Use of Laboratory Animals*, 8th edition.

### Human plasma samples

We collected blood samples from 5 patients who underwent lung transplantation in this study. Organ procurement adhered to the standard protocol outlined by the China Organ Transplant Response System (COTRS). Importantly, none of the organs utilized for lung transplantation in our study were procured from executed prisoners. Blood samples before surgery and 4 and 24 h after transplantation were collected. Subsequently, the whole blood underwent centrifugation at 1,000*g* for 10 min at 4 °C. The plasma supernatant was then harvested and subjected to a second centrifugation at 2,000*g* for 5 min at 4 °C. Following aliquoting, the plasma was frozen at −80 °C until the extraction of metabolites. Details of all participants are provided in Table S1.

### Animals

Eight- to 10-week-old male C57BL/6 wild-type (WT) and *Alox12*-KO mice were purchased from GemPharmatech in Jiangsu, China. In orthotopic left lung transplantation experiment, male inbred Sprague–Dawley (SD) rats, aged 8 to 12 weeks and weighing between 250 and 300 g, were procured from Charles River (Beijing, China) to serve as both donors and recipients. All animals were kept in a pathogen-free environment at the Shanghai Pulmonary Hospital Animal Facility (Shanghai, China), with unrestricted access to water and standard laboratory food

### Mouse left hilar clamp model for lung IRI

A mouse model of lung IRI was established as previously reported [43]. Briefly, mice underwent left hilar clamping for 1 h followed by a 3-h release period to induce lung IRI, a widely recognized murine model for studying PGD as outlined in the consensus statement [32]. The details are described in Supplementary Materials and Methods.

### Orthotopic left lung transplantation rat model for lung IRI

As we previously described, we established the orthotopic left lung transplantation rat model using the cuff technique [33]. Briefly, the donor lung was harvested and stored at 4 °C in Celsior solution for 18 h to induce prolonged cold ischemia, and then transplanted into the recipient rat to induce 2-h reperfusion before euthanasian. Celsior solution was chosen as the preservation solution to improve pulmonary graft preservation and avoid irreversible or fatal IRI to the animals, since a previous report has demonstrated its potential advantages in the context of extreme cold ischemia time [44]. The details are described in Supplementary Materials and Methods.

### Lung histology

After reperfusion, left lung tissues were collected and fixed in 4% paraformaldehyde for 48 h, after which they were embedded in paraffin. The entire lung was sequentially sliced and subjected to H&E staining. The lung injury score was calculated according to the American Thoracic Society workshop report [45]. Ten randomly chosen fields in each mouse were evaluated.

### Immunofluorescence of lung tissue

For immunofluorescence staining, the samples were incubated with rabbit anti-histone H3 (citrulline R2 + R8 + R17) (1:100, Abcam, ab5103) and rat anti-Ly6G (1:200, 551459, BD Pharmingen) overnight at 4 °C. Nuclei were stained with 4′,6-diamidino-2-phenylindole (DAPI) (1:1,000).

### Measurement of pulmonary function for mice and rats

At the end of scheduled reperfusion, the animals underwent anesthesia and tracheostomy and were positioned in a plethysmograph (EMMS, Hants, UK) to measure pulmonary resistance and compliance according to the manufacturer’s instructions. The details are described in Supplementary Materials and Methods.

### Pulmonary microvascular permeability

Pulmonary microvascular permeability was measured by the Evans blue dye extravasation technique as previously reported [46]. The details are described in Supplementary Materials and Methods.

### Lung wet/dry ratio

After reperfusion, the left lung was collected, weighed, and subsequently dried at 60 °C until a consistent weight was attained. The wet-to-dry weight ratio of the lungs was calculated as an indicator of lung edema.

### Survival analysis

The survival experiment was performed as previously reported [20]. Following the reperfusion period, the animals were anesthetized, and the right hilum was clamped, causing the survival and gas exchange of the animals to rely solely on the left lung. Observation of the survival then persisted for 1 h.

### Pear’s DAB staining and iron assay

We utilized DAB-enhanced Perls’ staining to identify iron deposition in lung sections embedded in paraffin. The details are described in Supplementary Materials and Methods.

### Bronchoalveolar lavage

After reperfusion, the right hilum was ligated and the left lung underwent lavage with 0.5 ml of cold phosphate-buffered saline. The BALF was centrifuged at 4 °C, 500*g* for 5 min, and the supernatant was divided into aliquots and stored at −80 °C for subsequent analysis.

### Quantification of NETs

Quantification of NETs in BALF and plasma was performed by detecting the level of MPO-DNA complex via a sandwich ELISA as previously reported [47]. The details are described in Supplementary Materials and Methods.

### Nontargeted metabolomics

Nontargeted metabolomics was performed for mice lung tissues and human plasma samples. The details of nontargeted metabolomics are described in Supplementary Materials and Methods.

### scRNA-Seq analysis

Single-cell suspensions from left lungs in the sham and IRI groups were prepared as previously reported [48]. Briefly, lung tissues were mechanically minced using fine scissors into RPMI1640 containing 480 U/ml collagenase type I (Thermo Fisher Scientific), 50 U/ml dispase (Roche), and 0.33 U/ml DNase (Roche) and incubated for 45 min at 37 °C with gentle shaking every 5 to 10 min. Cell solution was filtered through 70-μm cell strainer (Biosharp). Erythrocytes were removed with erythrocyte lysates. Cell suspensions were stained with anti-mouse CD45 antibody (Thermo Fisher Scientific, 45-0451-82) and LIVE/DEAD stain kit (Invitrogen, L34973). CD45-negative cells were sorted with a BD AriaII with over 95% purity.

The details of single-cell library construction and sequencing, and bioinformatic analysis of scRNA-Seq data are described in Supplementary Materials and Methods.

### RNA sequencing

Total RNA was extracted from HPMVECs or lung tissues by Trizol reagent. cDNAs of reverse transcription were sequenced on an Illumina Novaseq 6000 platform. The details of RNA-Seq analysis are described in Supplementary Materials and Methods.

### Cell culture and treatment

The HPMVEC cells were purchased from Fuheng Biology (Shanghai, China), and the HUVEC cells were purchased from Procell (Wuhan, China). The endothelial cells were cultured as we previously reported [49]. *ALOX12* siRNA was designed and chemically synthesized by OBIO Tech (Shanghai, China). The sequences of the si-*ALOX12* were as follows: GAAGCAUCGAGAGAAGGAACUTT (sense), AGUUCCUUCUCUCGAUGCUUCTT (antisense); GGAAGAGCUUCAGGCUCAACUTT (sense), AGUUGAGCCUGAAGCUCUUCCTT (antisense); GCUACACCAUGGAAAUCAACATT (sense), UGUUGAUUUCCAUGGUGUAGCTT (antisense). The impact of gene silencing was confirmed through Western blot analysis.

### In vitro hypoxia–reoxygenation model

Cells were placed in a sealed hypoxia chamber (Billups-Rothenberg, Del Mar, CA) and subjected to purging with 95% N_2_ and 5% CO_2_ at a flow rate of 40 l/min for 4 min to induce hypoxia following the manufacturer’s instructions. Then, the chamber was placed in a cell culture incubator for 12 h, after which it was opened for 4-h reoxygenation.

### GSH/GSSG, MDA, and LDH assay

The levels of GSH and GSSG in tissue or cell lysates were assessed with a GSSG/GSH Quantification Kit (Dojindo, G263). The level of MDA in tissue or cell lysates was also assessed with an MDA Assay Kit (Dojindo, M496). The level of lactate dehydrogenase (LDH) in cell culture supernatants was assessed with a Cytotoxicity LDH Assay Kit (Dojindo, CK12).

### The detection of Fe^2+^, ROS, and lipid peroxidation in cells

HPMVEC cells and HUVEC cells were stained with FerroOrange (Dojindo, F374), Liperfluo (Dojindo, L248), and ROS Assay Kit Highly Sensitive DCFH-DA (Dojindo, R252) to detect the levels of Fe^2+^, lipid peroxidation, and ROS by confocal microscopy. The signals were also quantified by flow cytometry (BD Canto II), and data were analyzed by FlowJo software v.10.8.1.

### Transmission electron microscopy

Following a 48-h incubation period, lung tissues were treated with 2% glutaraldehyde/0.1 M phosphate buffer (pH 7.4), dehydrated using ethanol, and then embedded in ultra-thin sections. Then, the ultra-thin sections were treated with uranyl acetate and lead citrate before being examined with the Hitachi HT7800 transmission electron microscope.

### Neutrophil isolation

Human neutrophils were obtained from healthy donors through density gradient centrifugation with PolymorphPrep (Axis Shield, Oslo, Norway). In short, 5 ml of blood was overlaid onto 5 ml of PolymorphPrep solution and centrifuged at 500*g* for 35 min at 20 °C without applying the brake. Following centrifugation, the layer containing neutrophils was gathered and rinsed with 50% Hanks’ balanced salt solution (HBSS) lacking Ca^2+^ and Mg^2+^. Neutrophils were harvested by centrifugation at 350*g* for 10 min and washed twice with HBSS. Contaminating erythrocytes were removed with erythrocyte lysates (Biosharp, BL503B). The purity and activity (>95%) of human neutrophils were confirmed by flow cytometry. Neutrophils (1 × 10^6^) were resuspended with different conditioned medium from endothelial cells and cultured in a humidified incubator (5% CO_2_) at 37 °C for 4 h. To visualize NET formation, neutrophils were stained with DAPI, anti-MPO (Invitrogen, PA5-16672), and anti-H3Cit (Abcam, ab5103). Images were obtained by laser scanning confocal microscopy.

### Flow cytometry

To measure the membrane expression of TLR4, neutrophils were initially treated with Fc block, followed by a 30-min surface staining using phycoerythrin anti-human TLR4 antibody (BioLegend, 312805). To measure the percentage of neutrophils in lung tissues, single-cell suspensions from left lungs were stained with LIVE/DEAD stain kit (Invitrogen, L34973), anti-mouse CD11b antibody (Thermo Fisher Scientific, 25-0112-82), and anti-mouse Ly6G antibody (BioLegend, 127605). Flow cytometry acquisition was performed on BD Canto II, and data were then analyzed with FlowJo software v.10.8.1.

### Statistical analysis

The statistical difference between 2 groups was determined using an independent *t* test for data with a normal distribution and a Mann–Whitney test for data that did not follow a normal distribution. For comparisons involving more than 2 groups, data with a normal distribution were analyzed using one-way analysis of variance (ANOVA) followed by Tukey’s multiple comparison test, while data not following a normal distribution were assessed using the post hoc test with Dunnett’s T3. Comparison of survival curves was analyzed by log-rank (Mantel–Cox) test. Statistical analyses were conducted using GraphPad Prism (version 10.1.2) software. *P* < 0.05 was considered statistically significant.

## Data Availability

The RNA-Seq raw data have been deposited in the Genome Sequence Archive (GSA) database of the National Genomics Data Center (NGDC, https://bigd.big.ac.cn/) under accession numbers CRA014486 and HRA006572. The scRNA-Seq raw data have been deposited in CNGB Sequence Archive (CNSA) of China National GeneBank DataBase (CNGBdb, https://db.cngb.org/cnsa/) with accession number CNP0005275.

## Supplementary Materials

Supplementary 1 Materials and Methods Figs. S1 to S7 Tables S1 and S2 References

## References

[1] Gelman AE, Fisher AJ, Huang HJ, Baz MA, Shaver CM, Egan TM, Mulligan MS. Report of the ISHLT Working Group on Primary Lung Graft Dysfunction Part III: Mechanisms: A 2016 Consensus Group Statement of the International Society for Heart and Lung Transplantation. J Heart Lung Transplant. 2017;36(10):1114–1120. 28818404 10.1016/j.healun.2017.07.014 PMC5724959

[2] Diamond JM, Arcasoy S, Kennedy CC, Eberlein M, Singer JP, Patterson GM, Edelman JD, Dhillon G, Pena T, Kawut SM, et al. Report of the International Society for Heart and Lung Transplantation Working Group on Primary Lung Graft Dysfunction, part II: Epidemiology, risk factors, and outcomes—A 2016 Consensus Group statement of the International Society for Heart and Lung Transplantation. J Heart Lung Transplant. 2017;36(10):1104–1113. 28802530 10.1016/j.healun.2017.07.020

[3] Wong A, Liu M. Inflammatory responses in lungs from donation after brain death: Mechanisms and potential therapeutic targets. J Heart Lung Transplant. 2021;40(9):890–896. 34167864 10.1016/j.healun.2021.03.010

[4] Stockwell BR. Ferroptosis turns 10: Emerging mechanisms, physiological functions, and therapeutic applications. Cell. 2022;185(14):2401–2421. 35803244 10.1016/j.cell.2022.06.003 PMC9273022

[5] Yamada N, Karasawa T, Wakiya T, Sadatomo A, Ito H, Kamata R, Watanabe S, Komada T, Kimura H, Sanada Y, et al. Iron overload as a risk factor for hepatic ischemia-reperfusion injury in liver transplantation: Potential role of ferroptosis. Am J Transplant. 2020;20(6):1606–1618. 31909544 10.1111/ajt.15773

[6] Cai W, Liu L, Shi X, Liu Y, Wang J, Fang X, Chen Z, Ai D, Zhu Y, Zhang X. Alox15/15-HpETE aggravates myocardial ischemia-reperfusion injury by promoting cardiomyocyte ferroptosis. Circulation. 2023;147(19):1444–1460. 36987924 10.1161/CIRCULATIONAHA.122.060257

[7] Meng H, Yu Y, Xie E, Wu Q, Yin X, Zhao B, Min J, Wang F. Hepatic HDAC3 regulates systemic iron homeostasis and ferroptosis via the Hippo signaling pathway. Research. 2023;6:0281. 38034086 10.34133/research.0281 PMC10687581

[8] Lee JY, Nam M, Son HY, Hyun K, Jang SY, Kim JW, Kim MW, Jung Y, Jang E, Yoon SJ, et al. Polyunsaturated fatty acid biosynthesis pathway determines ferroptosis sensitivity in gastric cancer. Proc Natl Acad Sci USA. 2020;117(51):32433–32442. 33288688 10.1073/pnas.2006828117 PMC7768719

[9] Mashima R, Okuyama T. The role of lipoxygenases in pathophysiology; new insights and future perspectives. Redox Biol. 2015;6:297–310. 26298204 10.1016/j.redox.2015.08.006 PMC4556770

[10] Chu B, Kon N, Chen D, Li T, Liu T, Jiang L, Song S, Tavana O, Gu W. ALOX12 is required for p53-mediated tumour suppression through a distinct ferroptosis pathway. Nat Cell Biol. 2019;21:579–591. 30962574 10.1038/s41556-019-0305-6 PMC6624840

[11] Zhong C, Yang J, Zhang Y, Fan X, Fan Y, Hua N, Li D, Jin S, Li Y, Chen P, et al. TRPM2 mediates hepatic ischemia-reperfusion injury via ca(2+)-induced mitochondrial lipid peroxidation through increasing ALOX12 expression. Research. 2023;6:0159. 37275121 10.34133/research.0159 PMC10232356

[12] Capuzzimati M, Hough O, Liu M. Cell death and ischemia-reperfusion injury in lung transplantation. J Heart Lung Transplant. 2022;41(8):1003–1013. 35710485 10.1016/j.healun.2022.05.013

[13] McDonald B, Pittman K, Menezes GB, Hirota SA, Slaba I, Waterhouse CC, Beck PL, Muruve DA, Kubes P. Intravascular danger signals guide neutrophils to sites of sterile inflammation. Science. 2010;330(6002):362–366. 20947763 10.1126/science.1195491

[14] Papayannopoulos V. Neutrophil extracellular traps in immunity and disease. Nat Rev Immunol. 2018;18(2):134–147. 28990587 10.1038/nri.2017.105

[15] Sayah DM, Mallavia B, Liu F, Ortiz-Muñoz G, Caudrillier A, DerHovanessian A, Ross DJ, Lynch JP 3rd, Saggar R, Ardehali A, et al. Neutrophil extracellular traps are pathogenic in primary graft dysfunction after lung transplantation. Am J Respir Crit Care Med. 2015;191(4):455–463. 25485813 10.1164/rccm.201406-1086OC PMC4351593

[16] Scozzi D, Wang X, Liao F, Liu Z, Zhu J, Pugh K, Ibrahim M, Hsiao H-M, Miller MJ, Yizhan G, et al. Neutrophil extracellular trap fragments stimulate innate immune responses that prevent lung transplant tolerance. Am J Transplant. 2019;19(4):1011–1023. 30378766 10.1111/ajt.15163 PMC6438629

[17] Caldarone L, Mariscal A, Sage A, Khan M, Juvet S, Martinu T, Zamel R, Cypel M, Liu M, Planiyar N, et al. Neutrophil extracellular traps in ex vivo lung perfusion perfusate predict the clinical outcome of lung transplant recipients. Eur Respir J. 2019;53(4):1801736. 30655281 10.1183/13993003.01736-2018

[18] Baciu C, Shin J, Hsin M, Cypel M, Keshavjee S, Liu M. Altered purine metabolism at reperfusion affects clinical outcome in lung transplantation. Thorax. 2023;78(3):249–257. 35450941 10.1136/thoraxjnl-2021-217498

[19] Reyfman PA, Walter JM, Joshi N, Anekalla KR, McQuattie-Pimentel AC, Chiu S, Fernandez R, Akbarpour M, Chen C-I, Ren Z, et al. Single-cell transcriptomic analysis of human lung provides insights into the pathobiology of pulmonary fibrosis. Am J Respir Crit Care Med. 2019;199(12):1517–1536. 30554520 10.1164/rccm.201712-2410OC PMC6580683

[20] Okada K, Fujita T, Minamoto K, Liao H, Naka Y, Pinsky DJ. Potentiation of endogenous fibrinolysis and rescue from lung ischemia/reperfusion injury in interleukin (IL)-10-reconstituted IL-10 null mice. J Biol Chem. 2000;275(28):21468–21476. 10806208 10.1074/jbc.M002682200

[21] Zheng Z, Chiu S, Akbarpour M, Sun H, Reyfman PA, Anekalla KR, Abdala-Valencia H, Edgren A, Li W, Kreisel D, et al. Donor pulmonary intravascular nonclassical monocytes recruit recipient neutrophils and mediate primary lung allograft dysfunction. Sci Transl Med. 2017;9(394):eaal4508. 28615357 10.1126/scitranslmed.aal4508 PMC5568853

[22] Xu Y, Li X, Cheng Y, Yang M, Wang R. Inhibition of ACSL4 attenuates ferroptotic damage after pulmonary ischemia-reperfusion. FASEB J. 2020;34(12):16262–16275. 33070393 10.1096/fj.202001758R

[23] Gong T, Liu L, Jiang W, Zhou R. DAMP-sensing receptors in sterile inflammation and inflammatory diseases. Nat Rev Immunol. 2020;20(2):95–112. 31558839 10.1038/s41577-019-0215-7

[24] Murao A, Aziz M, Wang H, Brenner M, Wang P. Release mechanisms of major DAMPs. Apoptosis. 2021;26(3–4):152–162. 33713214 10.1007/s10495-021-01663-3 PMC8016797

[25] Wen Q, Liu J, Kang R, Zhou B, Tang D. The release and activity of HMGB1 in ferroptosis. Biochem Biophys Res Commun. 2019;510(2):278–283. 30686534 10.1016/j.bbrc.2019.01.090

[26] Zhan Y, Ling Y, Deng Q, Qiu Y, Shen J, Lai H, Chen ZR, Huang CY, Lian LQ, Li X, et al. HMGB1-mediated neutrophil extracellular trap formation exacerbates intestinal ischemia/reperfusion-induced acute lung injury. J Immunol. 2022;208(4):968–978. 35063996 10.4049/jimmunol.2100593

[27] Hashimoto K, Cypel M, Juvet S, Saito T, Zamel R, Machuca TN, Hsin M, Kim H, Waddell TK, Liu M, et al. Higher M30 and high mobility group box 1 protein levels in ex vivo lung perfusate are associated with primary graft dysfunction after human lung transplantation. J Heart Lung Transplant. 2017;37(2):240–249. 10.1016/j.healun.2017.06.005 28689646

[28] Li W, Feng G, Gauthier JM, Lokshina I, Higashikubo R, Evans S, Liu X, Hassan A, Tanaka S, Cicka M, et al. Ferroptotic cell death and TLR4/Trif signaling initiate neutrophil recruitment after heart transplantation. J Clin Invest. 2019;129(6):2293–2304. 30830879 10.1172/JCI126428 PMC6546457

[29] Huang H, Tohme S, Al-Khafaji AB, Tai S, Loughran P, Chen L, Wang S, Kim J, Billiar T, Wang Y, et al. Damage-associated molecular pattern-activated neutrophil extracellular trap exacerbates sterile inflammatory liver injury. Hepatology. 2015;62(2):600–614. 25855125 10.1002/hep.27841 PMC4515210

[30] Luci D, Jameson JB, II, Yasgar A, Diaz G, Joshi N, Kantz A, Markham K, Perry S, Kuhn N, Yeung J, et al. *Discovery of ML355, a potent and selective inhibitor of human 12-lipoxygenase. Probe reports from the NIH Molecular Libraries Program*. Bethesda (MD): National Center for Biotechnology Information; 2010. 25506969

[31] Ma K, Xiao A, Park SH, Glenn L, Jackson L, Barot T, Weaver JR, Taylor-Fishwick DA, Luci DK, Maloney DJ, et al. 12-Lipoxygenase inhibitor improves functions of cytokine-treated human islets and type 2 diabetic islets. J Clin Endocrinol Metab. 2017;102(8):2789–2797. 28609824 10.1210/jc.2017-00267 PMC5546865

[32] Lama VN, Belperio JA, Christie JD, El-Chemaly S, Fishbein MC, Gelman AE, Hancock WW, Keshavjee S, Freisel D, Laubach VE, et al. Models of lung transplant research: A consensus statement from the National Heart, Lung, and Blood Institute workshop. JCI Insight. 2017;2(9):e93121. 28469087 10.1172/jci.insight.93121 PMC5414568

[33] Gao P, Li C, Ning Y, Wu J, Zhang P, Liu X, Su Y, Zhao D, Chen C. Improvement of surgical techniques for orthotopic single lung transplantation in rats. Ann Transl Med. 2022;10(12):673. 35845494 10.21037/atm-22-2018 PMC9279794

[34] Ma XH, Liu JH, Liu CY, Sun WY, Duan WJ, Wang G, Kurihara H, He RR, Li Y-F, Chen Y, et al. ALOX15-launched PUFA-phospholipids peroxidation increases the susceptibility of ferroptosis in ischemia-induced myocardial damage. Signal Transduct Target Ther. 2022;7(1):288. 35970840 10.1038/s41392-022-01090-z PMC9378747

[35] Zhang XJ, Cheng X, Yan ZZ, Fang J, Wang X, Wang W, Liu ZY, Shen LJ, Zhang P, Wang PX, et al. An ALOX12-12-HETE-GPR31 signaling axis is a key mediator of hepatic ischemia-reperfusion injury. Nat Med. 2018;24(1):73–83. 29227475 10.1038/nm.4451

[36] Zhang XJ, Liu X, Hu M, Zhao GJ, Sun D, Cheng X, Xiang H, Huang YP, Tian RF, Shen LJ, et al. Pharmacological inhibition of arachidonate 12-lipoxygenase ameliorates myocardial ischemia-reperfusion injury in multiple species. Cell Metab. 2021;33(10):2059–2075.e10. 34536344 10.1016/j.cmet.2021.08.014

[37] Zhao J, Li J, Wei D, Gao F, Yang X, Yue B, Xiong D, Liu M, Xu H, Hu C, et al. Liproxstatin-1 alleviates lung transplantation-induced cold ischemia-reperfusion injury by inhibiting ferroptosis. Transplantation. 2023;107(10):2190–2202. 37202851 10.1097/TP.0000000000004638

[38] Saren G, Wong A, Lu YB, Baciu C, Zhou W, Zamel R, Soltanieh S, Sugihara J, Liu M. Ischemia-reperfusion injury in a simulated lung transplant setting differentially regulates transcriptomic profiles between human lung endothelial and epithelial cells. Cells. 2021;10(10):2713. 34685693 10.3390/cells10102713 PMC8534993

[39] Mobbs JI, Black KA, Tran M, Burger WAC, Venugopal H, Holman TR, Holinstat M, Thal DM, Glukhova A. Cryo-EM structures of human arachidonate 12S-lipoxygenase bound to endogenous and exogenous inhibitors. Blood. 2023;142(14):1233–1242. 37506345 10.1182/blood.2023020441 PMC10579047

[40] Tsung A, Sahai R, Tanaka H, Nakao A, Fink MP, Lotze MT, Yang H, Li J, Tracey KJ, Geller DA, et al. The nuclear factor HMGB1 mediates hepatic injury after murine liver ischemia-reperfusion. J Exp Med. 2005;201(7):1135–1143. 15795240 10.1084/jem.20042614 PMC2213120

[41] Tadie JM, Bae HB, Jiang S, Park DW, Bell CP, Yang H, Pittet JF, Tracey K, Thannickal VJ, Abraham E, et al. HMGB1 promotes neutrophil extracellular trap formation through interactions with Toll-like receptor 4. Am J Physiol Lung Cell Mol Physiol. 2013;304(5):L342–L349. 23316068 10.1152/ajplung.00151.2012 PMC3602738

[42] Luo S, Liao C, Zhang L, Ling C, Zhang X, Xie P, Su G, Chen Z, Zhang L, Lai T, et al. METTL3-mediated m6A mRNA methylation regulates neutrophil activation through targeting TLR4 signaling. Cell Rep. 2023;42(3): Article 112259. 36920907 10.1016/j.celrep.2023.112259

[43] Charles EJ, Chordia MD, Zhao Y, Zhang Y, Mehaffey JH, Glover DK, Dimastromatteo J, Chancellor WZ, Sharma AK, Kron IL, et al. SPECT imaging of lung ischemia-reperfusion injury using [(99m)Tc]cFLFLF for molecular targeting of formyl peptide receptor 1. Am J Physiol Lung Cell Mol Physiol. 2020;318(2):L304–L313. 31800262 10.1152/ajplung.00220.2018 PMC7052676

[44] Sommer SP, Warnecke G, Hohlfeld JM, Gohrbandt B, Niedermeyer J, Kofidis T, Haverich A, Strüber M. Pulmonary preservation with LPD and Celsior solution in porcine lung transplantation after 24 h of cold ischemia. Eur J Cardiothorac Surg. 2004;26(1):151–157. 15200994 10.1016/j.ejcts.2004.02.019

[45] Matute-Bello G, Downey G, Moore BB, Groshong SD, Matthay MA, Slutsky AS, Kuebler WM, Acute Lung Injury in Animals Study Group. An official American Thoracic Society workshop report: Features and measurements of experimental acute lung injury in animals. Am J Respir Cell Mol Biol. 2011;44(5):725–738. 21531958 10.1165/rcmb.2009-0210ST PMC7328339

[46] Ito K, Shimada J, Kato D, Toda S, Takagi T, Naito Y, Yoshikawa T, Kitamura N. Protective effects of preischemic treatment with pioglitazone, a peroxisome proliferator-activated receptor-gamma ligand, on lung ischemia-reperfusion injury in rats. Eur J Cardiothorac Surg. 2004;25(4):530–536. 15037267 10.1016/j.ejcts.2003.12.017

[47] Sil P, Yoo DG, Floyd M, Gingerich A, Rada B. High throughput measurement of extracellular DNA release and quantitative NET formation in human neutrophils in vitro. J Vis Exp. 2016;(112):52779. 27404503 10.3791/52779 PMC4993224

[48] Zepp JA, Zacharias WJ, Frank DB, Cavanaugh CA, Zhou S, Morley MP, Morrisey EE. Distinct mesenchymal lineages and niches promote epithelial self-renewal and myofibrogenesis in the lung. Cell. 2017;170(6):1134–1148.e10. 28886382 10.1016/j.cell.2017.07.034 PMC5718193

[49] Qin H, Zhuang W, Liu X, Wu J, Li S, Wang Y, Liu X, Chen C, Zhang H. Targeting CXCR1 alleviates hyperoxia-induced lung injury through promoting glutamine metabolism. Cell Rep. 2023;42(7): Article 112745. 37405911 10.1016/j.celrep.2023.112745

