# Supplementary material for: Inhibition of ALOX12–12-HETE Alleviates Lung Ischemia–Reperfusion Injury by Reducing Endothelial Ferroptosis-Mediated Neutrophil Extracellular Trap Formation
Source: Research (Wash D C). 2024 Sep 12;7:0473. doi: 10.34133/research.0473 (PMC11391482; doi:10.34133/research.0473)
Supplement: Supplementary 1 — Materials and Methods Figs. S1 to S7 Tables S1 and S2 References [file research.0473.f1.docx]

**Supplementary Data**

**Supplementary Materials and Methods**

**Supplementary Figures S1-7 and Figure Legends**

**Supplementary Table S1-S2**

**Supplementary Materials and Methods**

**Mouse left hilar-clamp model for lung IRI**

After randomization to receive sham surgery or lung IRI, mice were subjected to anesthesia by intraperitoneal injection of sodium pentobarbital (50 mg/kg, Sinopharm, Beijing, China). Subsequently, orotracheal intubation was performed using a 20-G catheter, and a ventilator (KW-100-2, KEW Basis, Beijing, China) was connected with ambient air at a tidal volume of 0.8 ml and a respiratory rate of 120 breaths per minute. A heating pad was used to sustain their body temperature. After left thoracotomy in the fourth intercostal space, the left pulmonary artery, vein, and bronchus of mice in the IR group was clamped by a microvascular clamp. After one-hour ischemia, the microvascular was removed, the chest was closed, and mice were removed from the ventilator, and allowed for the 3-hour reperfusion period. After reperfusion, blood samples were promptly collected from the left ventricle for arterial blood gas analysis. Also, left lung tissues were harvested, and stored at －80 ℃ for further analysis. For mice treated with ML355 (MCE, HY-12341), mice were administered ML355 (3mg/kg) that are dissolved in a solution (DMSO: Solutol: PEG400: water; 5: 10: 20: 65) as previously reported[1] through tail vein injection before hilar clamp. In the vehicle control group, mice received an equal volume of vehicle solution. For some experiments, mice were intraperitoneally injected with Erastin (MCE, HY-15763) at 40mg/kg once every 3 days for 2 weeks or recombinant HMGB1 (R&D Systems, 690-HMB-050) at 50 ug/kg before surgery as previously reported[2-4].

**Orthotopic left lung transplantation rat model for lung IRI**

SD rats were used as both donors and recipients. After anesthesia, donor rats were intubated and ventilated with ambient air, maintaining a tide volume of 10 mL/kg, and a respiratory rate of 80/minute. Following a median laparosternotomy, the left auricle was incised, and the lungs underwent perfusion through the pulmonary trunk with 20 mL of 4 ℃ organ preservation Celsior solution (Genzyme Corp, Boston, MA). Subsequently, the heart and lungs were harvested and preserved in Celsior solution at 4 ℃ for 18 hours. Following that, the left lung was isolated, and the left bronchus, pulmonary artery and vein were each cuffed with 14-G, 16-G, and 18-G catheters, respectively. After anesthetization, a left thoracotomy was conducted for the recipient rat and the pulmonary bronchus, artery, and vein were separated and secured with microvascular clamps. After the anastomoses by tying each cuff with 5-0 silk ligatures, the clamp was released to start reperfusion. The chest cavity was closed, and recipients were extubated when spontaneous respiration resumed. After two-hour reperfusion, blood from graft pulmonary vein was collected for arterial blood gas analysis and graft lung tissues were harvested. In ML355 group, recipient rats were treated with ML355 (3mg/kg) via tail vein injection after being anesthetized.

**Measurement of** **pulmonary function for mice and rats**

At the end of scheduled reperfusion, the animals were anesthetized with sodium pentobarbital (50 mg/kg), and then tracheostomized with 18 G catheter and ventilated (rate 250 breaths/min and tidal volume 250 μl). After calibration, mice were monitored in a resistance and compliance plethysmograph with a pneumotachograph connected to a transducer (EMMS, Hants, UK, <http://www.electromedsys.com/pulmonary.html>). Instantaneous calculation of pulmonary resistance and compliance were obtained and were recorded for a 5-min period as previously reported[5, 6].

**Publicly available datasets analysis**

To investigate whether AA metabolism pathway was enriched and whether *ALOX12* expression was upregulated in other lung diseases, publicly available transcriptomic datasets of human lung tissues from patients with COVID-19 (GSE151764[7], GSE155241[8] and GSE182917[9]), lung fibrosis (GSE53845[10] and dataset from Reyman *et al*[11]) or sepsis-induced acute lung injury (GSE10474[12] and GSE66890[13]) were obtained from GEO datasets or the supplementary material of their publication. GSE151764 contains transcriptomic data of 34 tissues from 16 patients with COVID-19 and 9 normal lung tissues from 6 patients as control. GSE155241 contains transcriptomic data of 3 COVID-19 lung autopsies and 3 healthy lung autopsies as control. GSE182917 contains transcriptomic data of 11 COVID-19 lung tissues and 3 healthy lung tissues as control. GSE53845 contains transcriptomic data of 40 lung biopsies from patients with idiopathic pulmonary fibrosis (IPF) and 8 lung biopsies from patients without IPF as control. The dataset from Reyman *et al* contains transcriptomic data of lung biopsy tissue obtained from 8 lung explants from transplant recipients with pulmonary fibrosis and 14 donors before transplantation as control. Because the transcriptomic data of lung tissues from patients with acute lung injury is not available, transcriptomic data of whole blood was used instead. GSE10474 contains transcriptomic data of whole blood from 13 patients with sepsis-induced acute lung injury and 21 patients with sepsis alone as control. GSE66890 contains transcriptomic data of whole blood from 29 patients with sepsis-induced acute lung injury and 27 patients with sepsis alone as control.

**Pulmonary microvascular permeability**

Evans blue (30 mg/kg; Solarbio, G1810) was injected intravenously 30 minutes before sacrifice. The pulmonary vasculature was then perfused for 10 minutes with PBS to eliminate intravascular dye. The lungs were submerged in 5 ml of formamide (MACKLIN, F809511) and subjected to homogenization. The homogenate underwent an incubation at 37 ℃ for 24 h and was later centrifuged at 5000g for 30 min to extract the Evans blue. The optical density of the supernatant was measured at 620 nm. The concentration of EBD was determined based on a standard curve and reported as microgram/milligram wet lung weight.

**Pear’s DAB staining and iron assay**

Briefly, lung tissue sections were rinsed with PBS and incubated in Perls’ solution (5% potassium ferrocyanide/10% hydrochloric acid) for 1 h, followed by a 15 min incubation in DAB. Moreover, the level of Fe^2+^ in lung tissues and cells were measured by using an Iron Assay Kit (Dojindo, I291) following the manufacturer’s instructions.

**Quantification of NETs**

Briefly, 96-well high-binding capacity ELISA plates were incubated with 50ul/well anti-MPO (Invitrogen, PA5-16672) diluted 1:1000 in carbonate-bicarbonate buffer (PH 9.6) overnight at 4 ℃. Following the rinsing of wells with PBS and the application of a 5% BSA blocking solution, 50 μL of BALF or plasma was introduced into the wells and allowed to incubate for 2 hours at room temperature. Subsequently, the wells underwent another round of washing and were exposed to peroxidase-labeled anti-DNA monoclonal antibody (cell death detection ELISA kit; Roche, 11774425001) at a dilution of 1:100 in incubation buffer for 2 hours at room temperature. The plate was washed three times and incubated with the peroxidase substrate (ABTS) for 30 mins at 37 °C in the dark, the optical density (OD) was measured at 405 nm.

**Non-targeted metabolomics**

Briefly, Metabolites were extracted from 100 µL of plasma or100 mg of lung tissues. For plasma, 400 μL of -20 ℃ methanol was added and vortex for 1 min. The mixture was then centrifuged at 12,000 rpm and 4 °C for 10 min, and the supernatant was concentrated and dried. The sample was redissolved by 150 ul of 2-chloro-l-phenylalanine (4 ppm) solution prepared with 80% methanol water. Subsequently, the supernatant was passed through a 0.22 μm membrane for LC-MS detection. A quality control sample was created by combining 20 μL from each of the plasma samples. For mice lung tissues, 100 mg of tissues were ground at 50 Hz for 60 s twice with 1000μL tissue extract consisted of 75 % of 9:1 methanol: chloroform and 25% of H_2_O. After the ultrasound disruption, the mixture was centrifuged for 10 min at 12,000 rpm and 4 °C, and the supernatant was transferred, concentrated, and dried. The sample was redissolved by adding 200 μL of 4 ppm prepared with 50% acetonitrile solution, and the supernatant was passed through a 0.22 μm membrane and transferred for LC-MS detection.

The Vanquish UHPLC System (Thermo Fisher Scientific, USA) was utilized for LC analysis and the chromatography was conducted using ACQUITY UPLC ® HSS T3 (150×2.1 mm, 1.8 μm) (Waters, Milford, MA, USA). The Proteowizard software (v3.0.8789) was employed to convert the raw data into mzXML format. Subsequently, a data matrix, comprising the m/z ratio, retention time, and relative ratio of the peak area, was obtained. Metabolites were identified by matched against public databases and in-house metabolite library built by BioNovoGene Co. Ltd. with a mass accuracy of 20 ppm.

Metabolic pathway enrichment, partial least squares discrimination analysis (PLS-DA), and differential metabolites analysis were performed with the MetaboAnalyst web service ([www.metaboanalyst.ca](http://www.metaboanalyst.ca))[14].

**RNA-seq analysis**

Differentially expressed genes (DEGs) were identified by screening the normalized read count data using the R package “limma”. A threshold for gene expression log2-transformed fold change greater than 1 or less than -1, along with a significance level of p < 0.05, was utilized to identify the most DEGs. The Gene Ontology (GO) enrichment analysis and the KEGG pathway enrichment analysis were performed based on the upregulated and downregulated DEGs. The GSEA was also performed with software GSEA (v4.1.0) and MSigDB.

**Single-cell library construction and sequencing**

The purified single cells were then processed for single-cell RNA sequencing (scRNA-seq) using the DNBelab C Series HT Single-Cell RNA Library kit (MGI, 940-000047-00) in accordance with the manufacturer's protocol. Subsequently, all libraries underwent further sequencing on the DNBSEQ-T1&5 platform at the China National Gene Bank (CNGB).

**Bioinformatic Analysis of scRNA-Seq Data**

Raw sequencing reads were filtered and demultiplexed using PISA (v0.12b, https://github.com/shiquan/PISA). Reads were aligned to the reference genome GRCm38 (mm10) by STAR (v2.7.1a). Also, PISA was used to generate a cell versus gene UMI count matrix. Downstream analysis was performed using Seurat (4.3.0) in R (v4.1.1). Cells that had fewer than 500 genes, 1,000 unique molecular identifiers, and 10,000 reads were filtered. Cells with more than 10% mitochondrial gene counts were also excluded. DoubletFinder (v2.0.3, https://github.com/chris-mcginnis-ucsf/DoubletFinder) was used to remove doublets in each library using default parameters, and the 5% of cells similar to the pseudo-doublets were excluded.

The count matrix was normalized (‘NormalizeData’ function), and the top 3000 most variable genes were selected (‘FindVariableGene’ function). Merged data from each library was scaled (‘ScaleData’ function) and principal component analysis was performed (‘RunPCA’ function). UMAP (‘RunUMAP’ function) and the shared nearest neighbor (‘FindNeighbors’ function) was followed using harmony embeddings instead of PCA. Finally, clustering (‘FindClusters’ function) was performed. We used ‘FindAllMarkers’ and ‘FindMarkers’ function in Seurat with default parameters to perform DEG analysis between clusters and groups.

**Lung endothelial cells sorting**

After preparing single-cell suspensions from the left lungs of WT or Alox12-KO mice subjected to either sham surgery or IRI, lung endothelial cells (CD45^-^CD31^+)^ were sorted by fluorescence activated cell sorting (FACS), as previously reported[15]. Briefly, cell suspensions were stained with anti-mouse CD45 antibody (ThermoFisher, 45-0451-82) and anti-mouse CD31 antibody (Biolegend, 160211). CD45^-^CD31^+^ cells were sorted with a BD AriaII with over 95% purity.

**ELISA**

The levels of IL-6, CXCL1, TNF-α, and G-CSF in BALF and plasma were detected by ELISA kits (Solarbio, SEKM-0007, 0046, 0034 and 0040) according to the manufacturer’s instructions. The level of 12-HETE was also quantified using ELISA kits (Enzo Life Science, ADI-900-050). The level of HMGB1 in BALF or cell supernatant was detected by ELISA kits (Elabscience, E-EL-M0676c and E-EL-M0676c).

**RT-PCR analysis**

The extraction of total RNA was carried out using Trizol reagent, followed by reverse transcription into cDNA utilizing a TaKaRa PrimeScript RT reagent kit (TaKaRa). Real-time PCR was conducted on a Roche Light Cycler 480 (Roche) using SYBR Green PCR Master Mix (TaKaRa). The levels of mRNA expression were calculated using the 2^-△△Ct^ method and normalized to GADPH gene. The primers used were summarized in tables S2.

**Western blot analysis**

Protein extraction from lung tissues or cells was carried out using RIPA buffer (Beyotime, China) supplemented with protease and phosphatase inhibitors on ice. Protein extracts were subjected to sodium dodecyl sulfate-polyacrylamide gel electrophoresis, transferred to a polyvinylidene difluoride membrane, and incubated with the following primary antibodies: anti–β-actin (1:4000, Abcam, ab8227), anti-ALOX12 (1:200, Santa Cruz, sc-365194), anti-GPX4(1:1000, Abcam, ab125066), anti-TLR4 (1:1000, Santa Cruz, sc-293072) and anti-MyD88 (1:500, Abcam, ab2064). Subsequently, the membranes were incubated with secondary antibodies (CST,7076,7074). The signals were visualized by enhanced chemiluminescence (Bio-Rad, USA). Quantification of western blot analysis was measured by ImageJ software (version 1.34). All experiments were performed in triplicate.

**Reference**

1. Zhang XJ, Cheng X, Yan ZZ, Fang J, Wang X, Wang W, et al. An ALOX12-12-HETE-GPR31 signaling axis is a key mediator of hepatic ischemia-reperfusion injury. Nature medicine. 2018; 24: 73-83.

2. Li Y, Zeng X, Lu D, Yin M, Shan M, Gao Y. Erastin induces ferroptosis via ferroportin-mediated iron accumulation in endometriosis. Human reproduction (Oxford, England). 2021; 36: 951-64.

3. Li Y, Cao Y, Xiao J, Shang J, Tan Q, Ping F, et al. Inhibitor of apoptosis-stimulating protein of p53 inhibits ferroptosis and alleviates intestinal ischemia/reperfusion-induced acute lung injury. Cell death and differentiation. 2020; 27: 2635-50.

4. Zhan Y, Ling Y, Deng Q, Qiu Y, Shen J, Lai H, et al. HMGB1-Mediated Neutrophil Extracellular Trap Formation Exacerbates Intestinal Ischemia/Reperfusion-Induced Acute Lung Injury. J Immunol. 2022; 208: 968-78.

5. Williams AS, Leung SY, Nath P, Khorasani NM, Bhavsar P, Issa R, et al. Role of TLR2, TLR4, and MyD88 in murine ozone-induced airway hyperresponsiveness and neutrophilia. Journal of applied physiology (Bethesda, Md : 1985). 2007; 103: 1189-95.

6. Müller T, Grimm M, De Vieira R, Cicko S, Dürk T, Sorichter S, et al. Local administration of uridine suppresses the cardinal features of asthmatic airway inflammation. Clinical & Experimental Allergy. 2010; 40: 1552-60.

7. Nienhold R, Ciani Y, Koelzer VH, Tzankov A, Haslbauer JD, Menter T, et al. Two distinct immunopathological profiles in autopsy lungs of COVID-19. Nature communications. 2020; 11: 5086.

8. Han Y, Duan X, Yang L, Nilsson-Payant BE, Wang P, Duan F, et al. Identification of SARS-CoV-2 inhibitors using lung and colonic organoids. Nature. 2021; 589: 270-5.

9. Wu H, He P, Ren Y, Xiao S, Wang W, Liu Z, et al. Postmortem high-dimensional immune profiling of severe COVID-19 patients reveals distinct patterns of immunosuppression and immunoactivation. Nature communications. 2022; 13: 269.

10. DePianto DJ, Chandriani S, Abbas AR, Jia G, N'Diaye EN, Caplazi P, et al. Heterogeneous gene expression signatures correspond to distinct lung pathologies and biomarkers of disease severity in idiopathic pulmonary fibrosis. Thorax. 2015; 70: 48-56.

11. Reyfman PA, Walter JM, Joshi N, Anekalla KR, McQuattie-Pimentel AC, Chiu S, et al. Single-Cell Transcriptomic Analysis of Human Lung Provides Insights into the Pathobiology of Pulmonary Fibrosis. American journal of respiratory and critical care medicine. 2019; 199: 1517-36.

12. Howrylak JA, Dolinay T, Lucht L, Wang Z, Christiani DC, Sethi JM, et al. Discovery of the gene signature for acute lung injury in patients with sepsis. Physiological genomics. 2009; 37: 133-9.

13. Kangelaris KN, Prakash A, Liu KD, Aouizerat B, Woodruff PG, Erle DJ, et al. Increased expression of neutrophil-related genes in patients with early sepsis-induced ARDS. American journal of physiology Lung cellular and molecular physiology. 2015; 308: L1102-13.

14. Pang Z, Xu L, Viau C, Lu Y, Salavati R, Basu N, et al. MetaboAnalystR 4.0: a unified LC-MS workflow for global metabolomics. Nature communications. 2024; 15: 3675.

15. Bian F, Lan YW, Zhao S, Deng Z, Shukla S, Acharya A, et al. Lung endothelial cells regulate pulmonary fibrosis through FOXF1/R-Ras signaling. Nature communications. 2023; 14: 2560.

**

**

**Fig. S1 Arachidonic acid (AA) metabolism pathway and *ALOX12* expression in patients with COVID-19, lung fibrosis and sepsis-induced acute lung injury. A-C** GESA of transcriptomic data in COVID-19 patients versus healthy controls showed that the AA metabolism pathway was not enriched in either group in datasets GSE151764 (A) and GSE155241 (B), while it was enriched in the control group in dataset GSE182917 (C). **D-F** *ALOX12* expression levels did not differ significantly between COVID-19 patients and healthy controls; n=16 in the control group and n=34 in the COVID-19 group for panel (D); n=3 in each group for panel (E); n=3 in the control group and n=11 in the COVID-19 group for panel (F). **G-H** In lung fibrosis, GSEA revealed enrichment of the AA metabolism pathway (G), with *ALOX12* downregulated as shown in the volcano plot (H) **I** GESA in dataset GSE53845 indicated no enrichment of the AA metabolism pathway in either the lung fibrosis or control group. **J-K** GESA in sepsis-induced acute lung injury patients versus controls showed the AA metabolism pathway was enriched in the acute lung injury group in dataset GSE10474 (J) but not in dataset GSE66890 (K). **L-N** *ALOX12* expression was lower in lung fibrosis patients compared to controls in dataset GSE53845 (L, n=8 in the control group and n=40 in the lung fibrosis group), but similar between sepsis-induced acute lung injury patients and controls in datasets GSE10474 (M, n=21 in the control group and n=13 in the acute lung injury group) and GSE66890 (N, n= n=27 in the control group and n=29 in the acute lung injury group). The data are presented as means ± SDs. Significance was examined by Mann-Whitney test in (**D-F, L-N**)

**

**

**Fig. S2. *Alox12* knockout inhibited IR-induced inflammation. A-H** *Alox12* deficiency inhibited the release of pro-inflammatory cytokines, including IL-6 (A-B), CXCL1 (C-D), TNF-α (E-F) and G-CSF(G-H) detected in both BALF and plasma after lung IR. n=6 in each group. The data are presented as means ± SDs. Significance was examined by One-Way ANOVA.





**Fig. S3. *Alox12* deficiency prevent neutrophil recruitment after lung IR. A-B** Representative flow cytometry images (A) and quantitative analysis (B) showed the percentage of neutrophils (CD11b^+^Ly6G^+^) in lung tissues increased after lung IR in WT mice, while *Alox12* deficiency prevented this recruitment. n=3 in each group. The data are presented as means ± SDs. Significance was examined by One-Way ANOVA.





**Fig. S4 Endothelial cells sorted from IRI lung tissues showed ferroptosis, which was inhibited in *Alox12*-KO mice. A** Primary endothelial cells (CD45^-^CD31^+^) were sorted from lung tissues using fluorescence activated cell sorting (FACS). **B-C** Endothelial cells sorted from IRI lung tissues exhibited increased lipid peroxidation, as measured by confocal microscopy (B) and flow cytometry (C), which was prevented by *Alox12* deficiency. Scale bar: 25μm. **D-F** Endothelial cells sorted from IRI lung tissues exhibited increased levels of cell injury (D), MDA (E) and a decreased GSH/GSSG ratio (F), all of which were inhibited in *Alox12*-KO mice. n=3 in each group. The data are presented as means ± SDs. Significance was examined by One-Way ANOVA in (**D-F**).





**Fig. S5. *ALOX12* knockdown in HUVEC cells inhibited hypoxia/ reoxygenation-induced ferroptosis *in vitro.* A** The verification of ALOX12 protein expression by Western blot in HPMVEC cells and HUVEC cells treated with siRNA. n=3. **B-G** *ALOX12* knockdown reduced HR-induced ferroptosis in HUVEC cells, as evidenced by lower levels of iron, lipid ROS, and total ROS, measured via confocal microscopy (B, D, F) and flow cytometry (C, E, G) using FerroOrange, Liperfluo, and ROS Assay Kit staining, respectively (n=3 per group; scale bars: 25 μm in B, D; 20 μm in F). **H** Immunoblotting showed HUVEC cells treated with Si-*ALOX12* prevented the downregulation of the GPX4 protein level induced by HR. **I** *ALOX12* knockdown protected against the HR-induced cell injury, as detected by LDH cytotoxicity assay; n=3 in each group. **J-K** Change in the markers of ferroptosis induced by HR, including the GSH/GSSG ratio (J) and the relative level of MDA (K) were prevented in HUVEC cells with *ALOX12* knockdown; n=3 in each group. The data are presented as means ± SDs. Significance was examined with One-Way ANOVA in (**C, E, G, I-K**).

**

Fig. S6. Erastin or** **rHMGB1 treatment reversed lung injury alleviation in *Alox12*-KO mice after IR. A-B** Blood gas analysis including PaO_2_ and PaCO_2_ (A), and pulmonary function measurements including airway compliance and airway resistance (B) showed that the improvements in *Alox12*-KO mice after IR were reversed by erastin or rHMGB1 treatment; n=7 in each group. **C-D** Erastin or rHMGB1 treatment reversed the protective effects in *Alox12*-KO mice after IR, as shown by increased pulmonary microvascular permeability (C, measured by Evans-Blue dye extravasation) and pulmonary edema (D, measured by lung wet/dry ratio); n=7 in each group. **E** Survival of mice depended solely upon the left lung with right hilum ligated showed the survival benefit in *Alox12*-KO mice after IR was reversed by the treatment with erastin or rHMGB1. n=10 in each group. The data are presented as means ± SDs. Significance was examined with One-Way ANOVA in (**A-D**) and Log-rank (Mantel-Cox) test in (**E**).





**Fig S7.** **Pharmacological inhibition of ALOX12 by ML355 reduced lung IRI and NET formation in both the hilar-clamp mouse model and the orthotopic left lung transplantation rat model. A-B** Blood gas analysis including PaO_2_ and PaCO_2_ (A), and pulmonary function analysis including airway compliance and airway resistance (B) revealed that ML355 preserved pulmonary function in the hilar-clamp mouse model; n=7 in each group. **C-E** Markers of ferroptosis, including Fe^2+^ levels (C), MDA levels (D), and GSH/GSSG ratio, indicated that ML355 attenuated ferroptosis in the hilar-clamp mouse model; n=5 in each group. **F** ML355 treatment inhibited ferroptosis induced by lung IRI, as shown by increased GPX4 protein levels in lung tissues. **G** ML355 treatment reduced NET formation after lung IR, as measured by MPO-DNA complex in the BALF (left) and plasma (right); n=5 in each group. **H-I** ML355 treatment reduces lung injury (H) and pulmonary edema (I), as measured by lung injury scores and wet/dry ratio, in the orthotopic left lung transplantation rat model; n=5 in each group. **J** Immunofluorescence staining for H3cit and Ly6G in lung tissues showed that ML355 reduced NET formation after prolonged IR. Scale bar: 50 μm. The data are presented as means ± SDs. Significance was examined with One-Way ANOVA in (**A-E, G-I**).

| **Table S1 Detailed demographics of the lung transplant recipients** | | | | | |
| --- | --- | --- | --- | --- | --- |
| Variables | Age | Gender | Diagnosis | Lung transplantation procedure | Cold ischemia time(min) |
| Patient 1 | 65 | female | ILD | right single lung transplant | 624 |
| Patient 2 | 68 | male | ILD | double lung transplant | 480 |
| Patient 3 | 62 | male | COPD | right single lung transplant | 502 |
| Patient 4 | 51 | male | bronchiectasis | double lung transplant | 540 |
| Patient 5 | 71 | male | ILD | left single lung transplant | 486 |
| ILD, interstitial lung disease; COPD, Chronic obstructive pulmonary disease. | | | | | |

| **Table S2 Primers for qPCR analysis** | | |
| --- | --- | --- |
| Gene | | Sequence 5'---3' |
| Alox12 | Forward | TCCCTCAACCTAGTGCGTTTG |
|  | Reverse | GTTGCAGCTCCAGTTTCGC |
| Gapdh | Forward | AGGTCGGTGTGAACGGATTTG |
|  | Reverse | TGTAGACCATGTAGTTGAGGTCA |
